# Induction of Potent Neutralizing Antibody Responses by a Designed Protein Nanoparticle Vaccine for Respiratory Syncytial Virus

**DOI:** 10.1016/j.cell.2019.01.046

**Published:** 2019-03-07

**Authors:** Jessica Marcandalli, Brooke Fiala, Sebastian Ols, Michela Perotti, Willem de van der Schueren, Joost Snijder, Edgar Hodge, Mark Benhaim, Rashmi Ravichandran, Lauren Carter, Will Sheffler, Livia Brunner, Maria Lawrenz, Patrice Dubois, Antonio Lanzavecchia, Federica Sallusto, Kelly K. Lee, David Veesler, Colin E. Correnti, Lance J. Stewart, David Baker, Karin Loré, Laurent Perez, Neil P. King

**Affiliations:** 1 Università della Svizzera italiana, Faculty of Biomedical Sciences, Institute for Research in Biomedicine, Bellinzona, Switzerland; 2 Department of Biochemistry, University of Washington, Seattle, WA, USA; 3 Institute for Protein Design, University of Washington, Seattle, WA, USA; 4 Department of Medicine Solna, Division of Immunology and Allergy, Karolinska Institutet, Stockholm, Sweden; 5 Center for Molecular Medicine, Karolinska Institutet, Stockholm, Sweden; 6 Institute of Microbiology, ETH Zürich, Switzerland; 7 Clinical Research Division, Fred Hutchinson Cancer Research Center, Seattle, WA, USA; 8 Department of Medicinal Chemistry, University of Washington, Seattle, WA, USA; 9 Vaccine Formulation Laboratory, University of Lausanne, Epalinges, Switzerland; 10 Vaccine Formulation Institute, Godalming, UK; 11 Biological Physics Structure and Design Program, University of Washington, Seattle, WA, USA; 12 Howard Hughes Medical Institute, University of Washington, Seattle, WA, USA; 13 European Virus Bioinformatics Center, Jena, Germany

**Keywords:** computational protein design, self-assembly, vaccines, respiratory syncytial virus, nanoparticles, neutralizing antibodies

## Abstract

Respiratory syncytial virus (RSV) is a worldwide public health concern for which no vaccine is available. Elucidation of the prefusion structure of the RSV F glycoprotein and its identification as the main target of neutralizing antibodies have provided new opportunities for development of an effective vaccine. Here, we describe the structure-based design of a self-assembling protein nanoparticle presenting a prefusion-stabilized variant of the F glycoprotein trimer (DS-Cav1) in a repetitive array on the nanoparticle exterior. The two-component nature of the nanoparticle scaffold enabled the production of highly ordered, monodisperse immunogens that display DS-Cav1 at controllable density. In mice and nonhuman primates, the full-valency nanoparticle immunogen displaying 20 DS-Cav1 trimers induced neutralizing antibody responses ∼10-fold higher than trimeric DS-Cav1. These results motivate continued development of this promising nanoparticle RSV vaccine candidate and establish computationally designed two-component nanoparticles as a robust and customizable platform for structure-based vaccine design.

## Highlights

• Design of a self-assembling protein immunogen displaying 20 copies of prefusion RSV F
• *In vitro* assembly yields highly ordered immunogens with tunable antigen density
• The nanoparticle immunogens induce potent neutralizing antibody responses
• Fusion of DS-Cav1 to the trimeric nanoparticle subunit stabilizes the antigen

## Graphical Abstract

**Figure:**
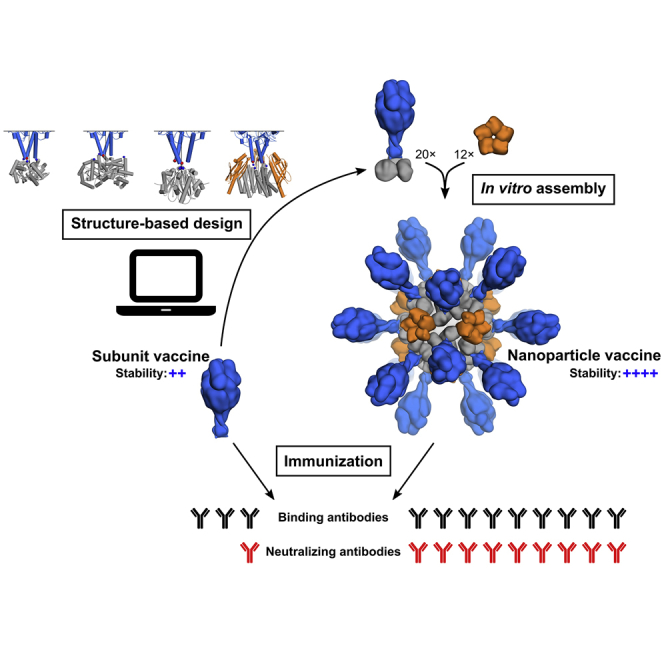

## Teaser

A computationally designed self-assembling nanoparticle that displays 20 copies of a trimeric viral protein induces potent neutralizing antibody responses.

## Introduction

Respiratory syncytial virus (RSV) is an enveloped RNA virus in the recently defined *Pneumoviridae* family (Afonso et al., 2016, Collins et al., 2013). RSV infection is extremely common, occurring in nearly all humans by the age of three and recurring throughout life (Glezen et al., 1986). Infection of healthy adults typically results in mild respiratory symptoms, but can be more serious in infants and older adults: RSV infection is second only to malaria as a cause of infant mortality worldwide (Lozano et al., 2012) and accounts for a substantial hospitalization burden in both age groups in developed countries (Hall et al., 2009, Widmer et al., 2012). Despite substantial effort, including a wide variety of vaccine candidates currently in preclinical or clinical development, a safe and effective vaccine for RSV has not yet been developed.

Of the three RSV surface proteins (F, G, and SH), F-specific antibodies account for the majority of neutralizing activity in the sera of infected humans (Magro et al., 2012, Ngwuta et al., 2015), and F is therefore the focus of many vaccine efforts. F is a trimeric type I fusion glycoprotein responsible for merging the viral membrane with cellular membranes, and, like many other viral fusion glycoproteins, it undergoes major structural rearrangements as it transitions from the prefusion to the postfusion state (Harrison, 2015). Due to the relative instability of the prefusion conformation, subunit vaccine candidates were until recently limited to the more stable postfusion structure (McLellan et al., 2011, Smith et al., 2012, Swanson et al., 2011). In clinical trials, these candidates have induced only modest increases in neutralizing antibodies (August et al., 2017, Langley et al., 2017), high levels of which correlate with a lower risk of infection (Falsey and Walsh, 1998, Glezen et al., 1986, Piedra et al., 2003).

Crystal structures of both the prefusion and postfusion forms of RSV F have provided key insights into antigenicity (McLellan et al., 2011, McLellan et al., 2013a, Swanson et al., 2011) and spurred the development of next-generation vaccine candidates based on prefusion F. The identification of a number of potent neutralizing antibodies that target epitopes specific to the prefusion structure (Beaumont et al., 2013, Corti et al., 2013, Gilman et al., 2016, Kwakkenbos et al., 2010, McLellan et al., 2013a, Wen et al., 2017), together with the observation that most neutralizing activity in human sera is prefusion-specific (Magro et al., 2012), predicted that F protein variants stabilized in the prefusion conformation would yield improved vaccine candidates. This prediction has been borne out in multiple studies of DS-Cav1, a prefusion-stabilized F antigen that has elicited significantly higher neutralizing antibody titers than postfusion F in naive mice and nonhuman primates (McLellan et al., 2013b) and bovine RSV-primed cattle (Steff et al., 2017) and is now in Phase I clinical trials (NCT03049488). Additional engineered prefusion RSV F antigens have shown similar improvements in eliciting neutralizing antibody responses and in some cases, exhibit improved physical and antigenic stability relative to DS-Cav1 (Joyce et al., 2016, Krarup et al., 2015, Palomo et al., 2016).

While the development of prefusion F antigens is a major breakthrough that has revitalized RSV vaccine development, the requirements for an effective vaccine remain unknown. Combining these antigens with orthogonal technologies to further increase the induction of neutralizing antibodies could improve the likelihood of protective efficacy in humans. It has long been known that presenting multiple copies of an antigen in a repetitive array can drive more robust humoral immune responses than “soluble” antigen. This effect is thought to derive mainly from stronger B cell activation through antigen-driven cross-linking of B cell receptors (BCRs) (Bachmann and Jennings, 2010), although potential effects on antigen trafficking and localization may also play a role (Irvine et al., 2013). Among several technologies explored in this context (reviewed in Gause et al., 2017, Irvine et al., 2015), self-assembling proteins are a powerful platform for multivalent antigen presentation (López-Sagaseta et al., 2015). They can form highly ordered, monodisperse structures that can be scalably manufactured, are naturally non-toxic, and offer seamless integration of protein antigens via genetic fusion. Recently, several self-assembling proteins such as ferritin, lumazine synthase, and encapsulin have been successfully used as scaffolds to present complex glycoprotein antigens derived from influenza hemagglutinin (Kanekiyo et al., 2013, Yassine et al., 2015), HIV envelope (Abbott et al., 2018, He et al., 2016, Jardine et al., 2015, McGuire et al., 2016, Sliepen et al., 2015), and Epstein-Barr virus (Kanekiyo et al., 2015). In all cases, immunogenicity of the antigen was increased by multivalent presentation, and in some cases, an epitope-focusing effect was observed in which potent neutralizing epitopes were preferentially targeted (Duan et al., 2018, Kanekiyo et al., 2015, Yassine et al., 2015).

However, structure-based design of nanoparticle immunogens has to date been limited by the small number of naturally occurring scaffolds available and the fact that their fundamental structural properties are fixed. Moreover, all of the widely used self-assembling scaffolds spontaneously self-assemble upon expression in a recombinant host cell. These constraints prevent the exploration of new structural and functional space in nanoparticle immunogen design. We recently described the development of general computational methods for designing novel self-assembling proteins with atomic-level accuracy (Bale et al., 2016, Hsia et al., 2016, King et al., 2012, King et al., 2014). The ability to create self-assembling proteins with customized structures offers new opportunities in structure-based vaccine design. Here, we combine the recent breakthroughs in prefusion F antigen design and custom protein nanomaterial design to produce a nanoparticle immunogen that induces ten-fold more potent neutralizing antibody responses than trimeric DS-Cav1, a leading clinical-stage RSV vaccine candidate.

## Results

### Design and Screening of DS-Cav1-Bearing Nanoparticle Components

**Figure 1:**
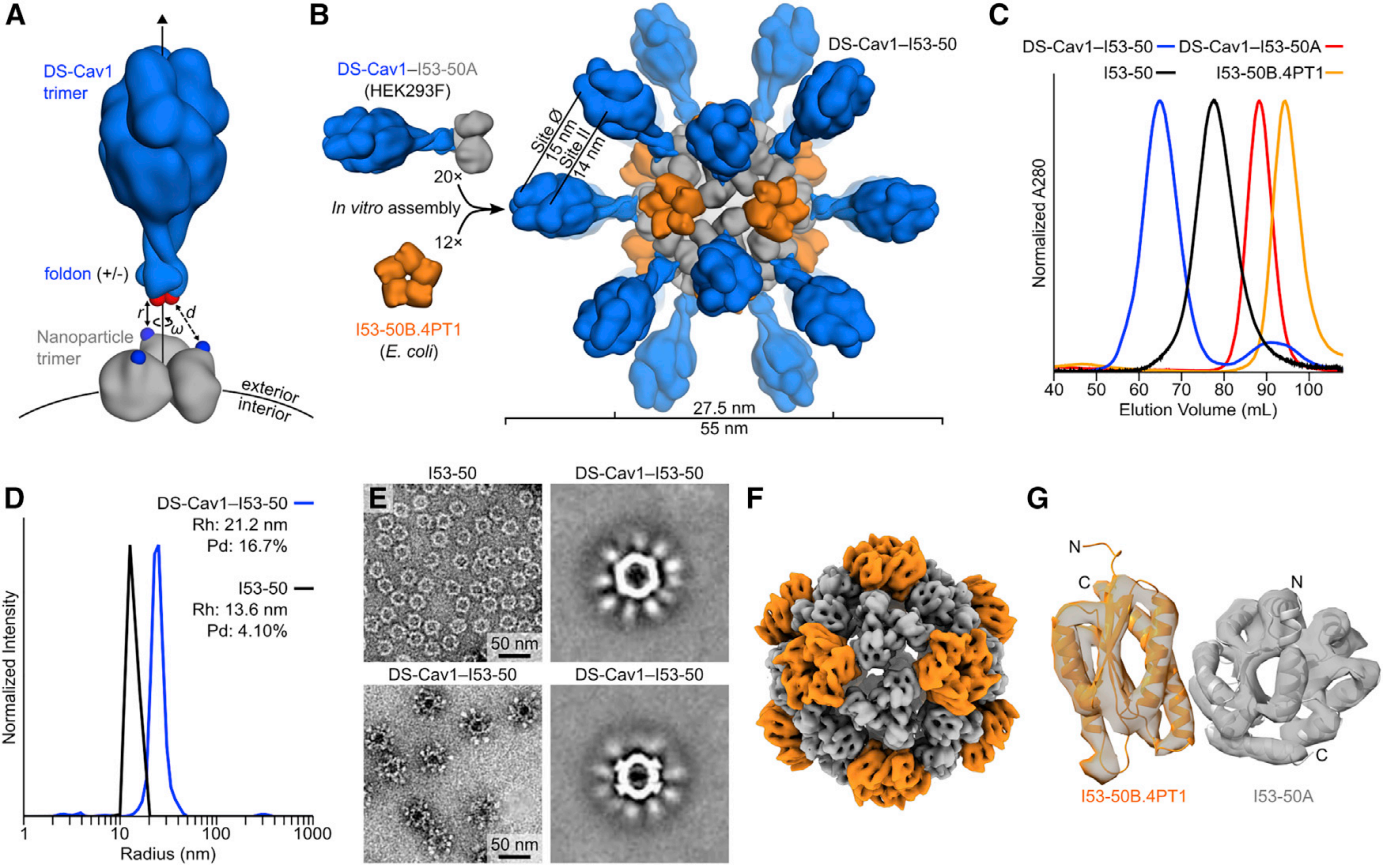
Design, *In Vitro* Assembly, and Structural Characterization of DS-Cav1-I53-50 (A) Schematic representation of the computational docking protocol used to identify nanoparticle components suitable for fusion to DS-Cav1. The C termini of the foldon and N termini of the nanoparticle trimer are shown as red and blue spheres, respectively, and the exterior and interior surfaces of the nanoparticle are depicted. (B) Structural model of DS-Cav1-I53-50 and schematic of the *in vitro* assembly process. Each nanoparticle comprises 20 trimeric and 12 pentameric building blocks for a total of 60 copies of each subunit. (C) Chromatograms of unassembled components and assembled nanoparticles on a Sephacryl S-500 HR 16/60 SEC column. (D) Dynamic light scattering of I53-50 and DS-Cav1-I53-50 nanoparticles. The hydrodynamic radius (Rh) and polydispersity (Pd) of each nanoparticle are indicated. (E) Negative stain EM of I53-50 and DS-Cav1-I53-50 nanoparticles. The two images on the right are averages of negatively stained particles. (F) Single-particle cryo-electron microscopy reconstruction of DS-Cav1-I53-50, determined at a resolution of 6.3 Å. The density is colored according to subunits as in (B). (G) Alignment of the icosahedral asymmetric unit of the I53-50 computational design model (Bale et al., 2016) to the cryo-EM reconstruction. The N and C termini of each subunit are indicated, and subunits are colored as in (B). See also Figure S1, Figure S2, Figure S3 and Tables S1 and S2.

**Figure S1:**
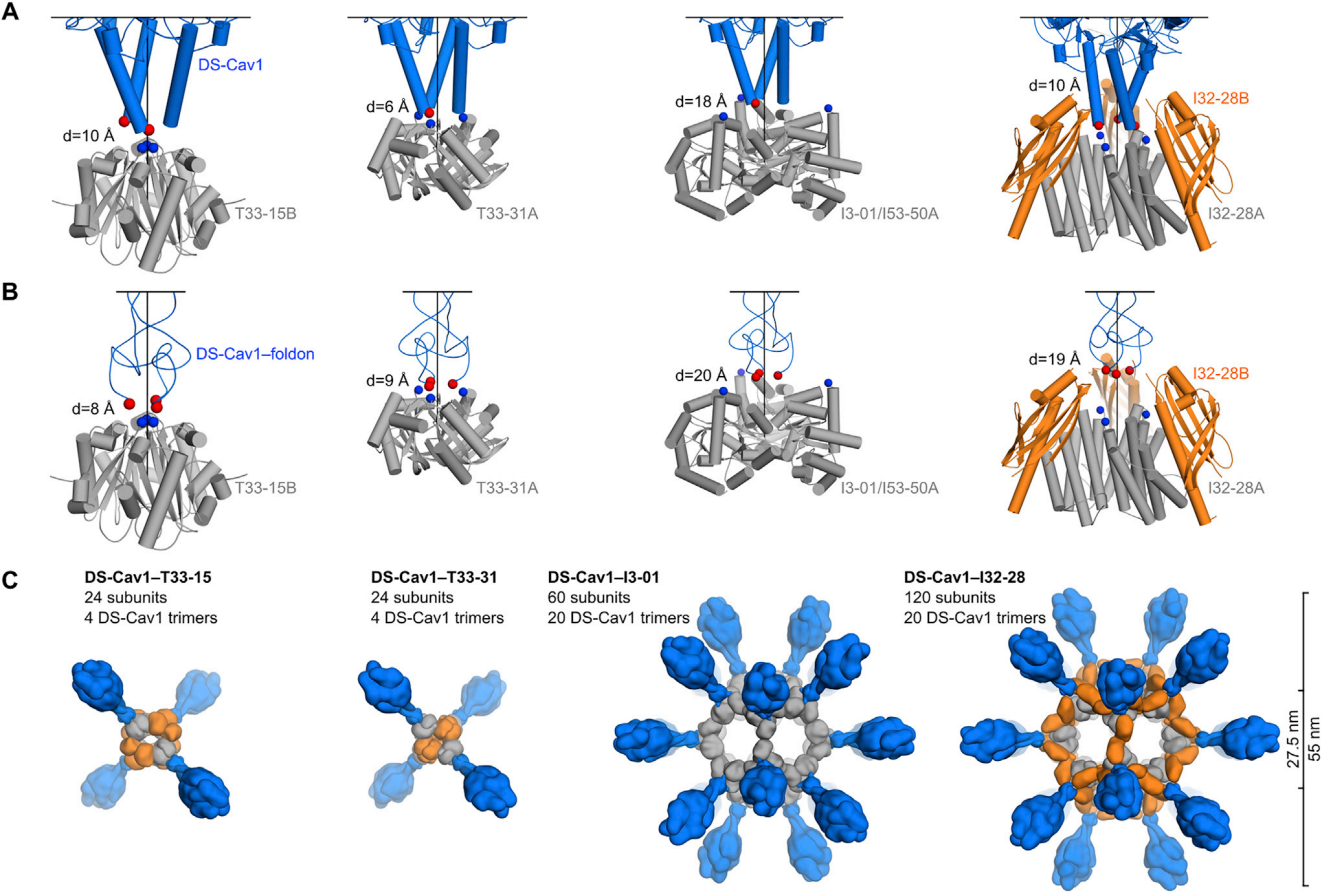
Computational Docking of Antigens and Nanoparticle Subunits and Nanoparticle Immunogen Design Models, Related to Figure 1 (A) Docking of DS-Cav1 (PDB ID 4mmu) to several trimeric nanoparticle components. The C termini of DS-Cav1 are indicated as red spheres, the N termini of the nanoparticle subunits as blue spheres. The linear distance between the termini (d) is given. All images are oriented such that the nanoparticle exterior is up. Because I3-01 and I53-50A were derived from the same trimeric building block, the docking was only performed once. Neighboring subunits of the dimeric component of I32-28 (I32-28B) were included during docking due to their extension outward beyond the I32-28A trimer. (B) Docking of DS-Cav1–foldon (PDB ID 4mmv) to several nanoparticle components. Images of the docking results are shown as in (A). (C) Structural models of prefusion F nanoparticle immunogens. Nanoparticle components are colored gray and orange; DS-Cav1-foldon is blue. All models are shown to scale; scale bar at right.

Our recently reported designed protein nanomaterials provided a library of potential scaffolds for multivalent presentation of DS-Cav1. We computationally docked crystal structures of DS-Cav1 with and without the C-terminal foldon domain (PDB: 4MMV, 4MMU) against the subset of trimeric building blocks from these materials that have N termini projecting outward. The 3-fold symmetry axes of each pair of trimers were aligned, and the distance between the C terminus of DS-Cav1 and the N terminus of the nanoparticle subunit was minimized while disallowing atomic clashes (Figure 1A). This procedure identified a subset of building blocks from nanoparticles of various sizes, symmetries, and numbers of subunits as promising candidates for genetic fusion to DS-Cav1 (Figures S1A and S1B). Trimeric building blocks from two two-component tetrahedral complexes, one one-component icosahedral complex, and two two-component icosahedral complexes were selected for experimental characterization. The tetrahedral assemblies would present 4 copies of the DS-Cav1 trimer (12 subunits) per particle, whereas the icosahedral assemblies would present 20 copies of the trimer (60 subunits), one along each pole of the 10 icosahedral 3-fold symmetry axes (Figures 1B and S1C).

**Figure S2:**
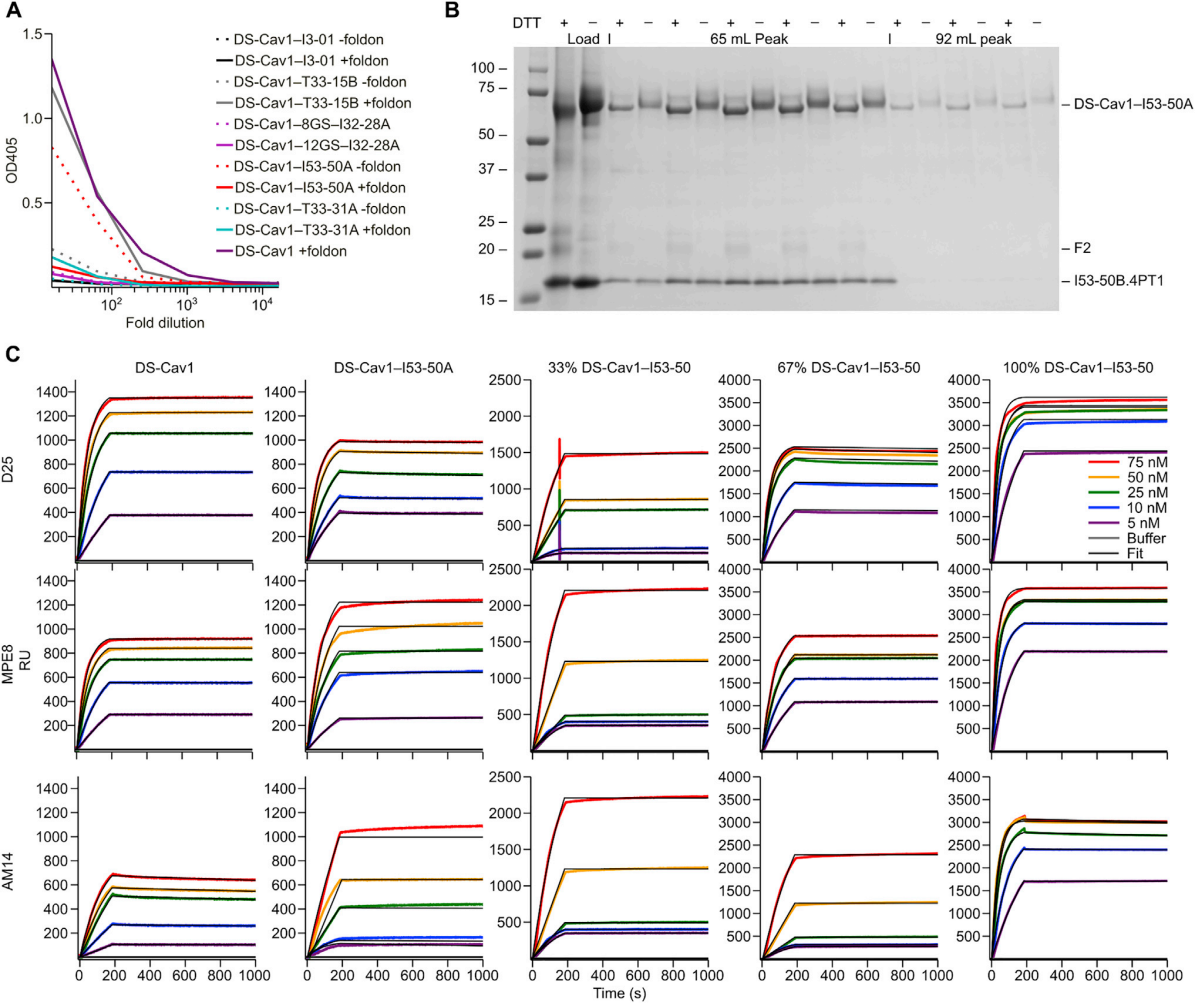
Screening, Biochemical, and Antigenic Characterization of DS-Cav1-Nanoparticle Subunit Fusions, Related to Figure 1 (A) ELISA-based screening of fusion proteins. Five trimeric nanoparticle components genetically fused to DS-Cav1 with or without the foldon (except I32-28A, which excluded foldon but used two linker lengths), were transfected into HEK293F cells. Cell culture supernatants were screened for secretion of the fusion proteins five days later by ELISA using D25 as the primary antibody. (B) SDS-PAGE of peak fractions from SEC of the DS-Cav1–I53-50 nanoparticle *in vitro* assembly reaction presented in Figure 1C. The 65 mL peak contained assembled nanoparticle and both components are visible by SDS-PAGE in the corresponding fractions. Residual DS-Cav1–I53-50A trimeric component is observed in the 92 mL peak. F2, the mature F2 subunit of DS-Cav1. Molecular weight markers are indicated in kilodaltons. (C) Antigenic characterization of DS-Cav1 and DS-Cav1–I53-50 nanoparticles by SPR. D25 (site Ø), MPE8 (site II/III), and AM14 (site V) mAbs were immobilized on a GLM chip (100 nM) through amine coupling (EDC/NHS chemistry) and a blank surface with no antibody was created under identical coupling conditions for use as a reference. Analyte proteins (trimeric DS-Cav1, trimeric DS-Cav1–I53-50A, and DS-Cav1–I53-50 nanoparticles), were injected at various concentrations (5 to 75 nM) in the different sensor channels. Data were processed using Proteon Manager software and double-referenced by subtraction of the blank surface and buffer-only injection before local fitting of the data using Langmuir fitting.

We transfected plasmids encoding each DS-Cav1 fusion protein (see Table S1 for amino acid sequences) into HEK293F cells and estimated protein yield in the culture media 5 days later by ELISA using the prefusion-specific monoclonal antibody (mAb) D25 (Figure S2A). With these data, we selected DS-Cav1-foldon fused to the trimeric component of I53-50 (“DS-Cav1-I53-50A”) for further characterization to maximize the antigen density that would result upon assembly of the icosahedral nanoparticle (Figure 1B) and to provide a close comparison to the foldon-containing trimeric DS-Cav1. I53-50 is a computationally designed two-component protein complex comprising 20 trimeric “A” components and 12 pentameric “B” components for a total of 120 subunits (Bale et al., 2016). A model of the DS-Cav1-I53-50 nanoparticle illustrates how the 20 DS-Cav1 trimers project outward ∼13 nm from the nanoparticle surface, with epitopes on neighboring DS-Cav1 trimers spaced ∼15 nm apart (Figure 1B).

### *In Vitro* Assembly and Structural Characterization of DS-Cav1-I53-50

**Figure S3:**
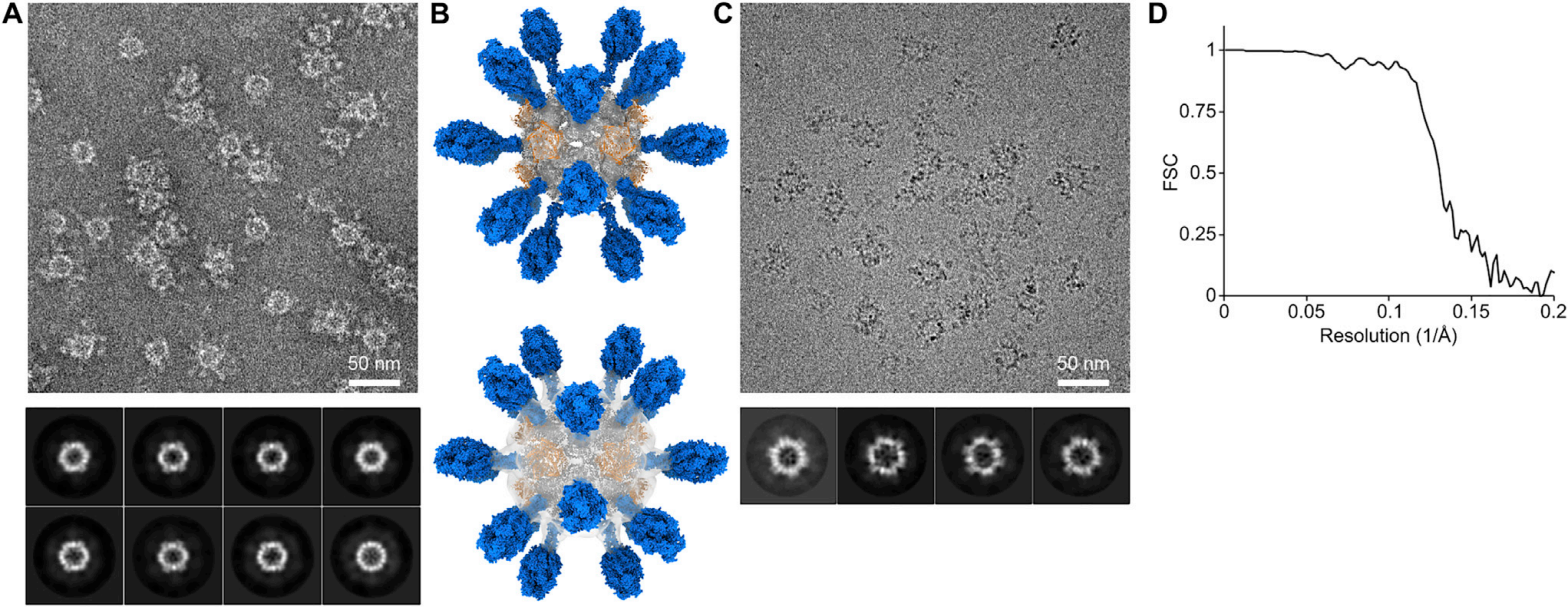
Characterization of DS-Cav1-I53-50 by Negative Stain and Cryoelectron Microscopy, Related to Figure 1 (A) Negative stain electron micrograph of DS-Cav1–I53-50 with associated 2D class averages obtained from 4300 particles. (B) Three-dimensional negative stain reconstruction of DS-Cav1–I53-50 at an estimated resolution of 20 Å. Two views are provided, with the electron density modeled at a level of 0.4 (top) and 0.04 (bottom). At lower signal to noise, some weak density is observed at the base of the displayed antigen. (C) Cryo-electron micrograph of DS-Cav1–I53-50 with associated 2D class averages obtained from 1600 particles. (D) FSC curve for the single-particle reconstruction of DS-Cav1–I53-50 shown in Figures 1E and 1F. Resolution at FSC = 0.143 is 6.3 Å.

We independently purified trimeric DS-Cav1-I53-50A from HEK293F supernatants and the pentameric component, I53-50B.4PT1, from *E. coli* cells by immobilized metal affinity chromatography and size exclusion chromatography (SEC). When analyzed by SEC on a Sephacryl S-500 column, each component eluted as a single peak near the elution volume expected for its oligomeric state (Figure 1C). In contrast, a mixture of the two purified components at a 1:1 molar ratio yielded a predominant peak at an early elution volume, suggesting efficient *in vitro* assembly to the target icosahedral structure. SDS-PAGE confirmed the presence of both components in the SEC peak, and a small amount of residual, unassembled trimeric component could also be detected after *in vitro* assembly (Figure S2B). The I53-50B.4PT1 pentamer mixed with unmodified I53-50A (the trimeric component lacking DS-Cav1) eluted slightly later, consistent with the smaller hydrodynamic radius expected in the absence of the displayed antigen. Dynamic light scattering (DLS) and negative stain electron microscopy (EM) of the SEC-purified DS-Cav1-bearing nanoparticles revealed a monodisperse population with a diameter of ∼44 nm, roughly in agreement with the design model and again larger than unmodified I53-50 (Figures 1D, 1E, and S3A).

The antigenic integrity of the DS-Cav1-I53-50 nanoparticles was evaluated by surface plasmon resonance (SPR) using the prefusion-specific mAbs D25, MPE8, and AM14 (Corti et al., 2013, Kwakkenbos et al., 2010). All three mAbs bind with similar kinetics to trimeric DS-Cav1 and trimeric DS-Cav1-I53-50A (Figure S2C), yielding similar calculated equilibrium dissociation constants (K_D_; Table S2). While the mAbs do not dissociate from DS-Cav1-I53-50 nanoparticles, likely due to increased avidity, the on-rates (k_on_) of the mAbs to the nanoparticle immunogen and the trimeric molecules are also similar (Table S2), supporting that assembly of the trimeric component into the nanoparticle does not affect antigenicity.

Although the displayed DS-Cav1 antigen was clearly visible upon imaging negatively stained DS-Cav1-I53-50 (Figures 1E and S3A), it was poorly resolved upon subsequent particle image averaging during data processing (Figures S3A and S3B), suggesting that the connection between DS-Cav1 and I53-50 is flexible, as expected given the extended linker connecting them. We vitrified DS-Cav1-I53-50 and determined a single particle cryo-electron microscopy reconstruction at a resolution of 6.3 Å in which only the I53-50 nanoparticle was resolved (Figures 1F, 1G, S3C, and S3D). Fitting the computationally designed model of I53-50 into the density supported the accuracy of the design and demonstrated that genetic fusion of DS-Cav1 to I53-50 did not affect the architecture of the two-component nanoparticle (Figure 1G).

Together, these data establish that two-component protein nanoparticles can display complex glycoprotein antigens and assemble *in vitro* to generate monodisperse immunogens with high efficiency.

### Physical Stabilization of DS-Cav1 by Fusion to I53-50A

**Figure 2:**
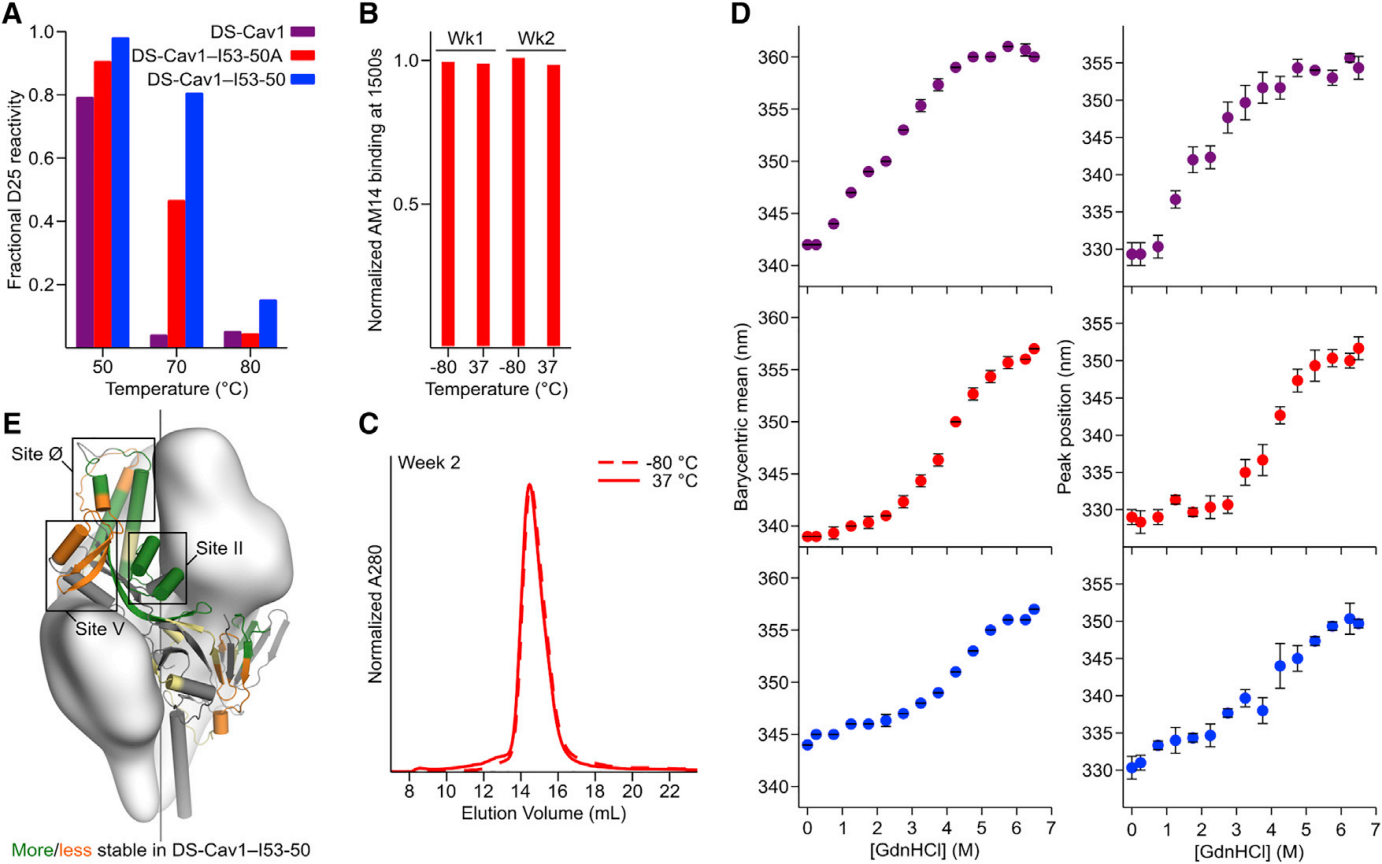
Physical Stabilization of DS-Cav1 by Fusion to I53-50A (A) Retention of D25 binding after thermal stress, measured by SPR. The y axis represents the amplitude of the SPR signal obtained from antigen incubated at the indicated temperature for 1 h relative to the signal after incubation at 20°C. Data from a representative experiment that was performed multiple times are shown. (B) Reactivity to AM14 mAb of trimeric DS-Cav1-I53-50A incubated for 1 or 2 weeks at −80°C or 37°C, measured by bio-layer interferometry. The amplitude of each signal after binding was complete is normalized to the week 1, −80°C sample. Data from a representative experiment that was performed twice are shown. (C) SEC chromatograms of trimeric DS-Cav1-I53-50A after incubation for 2 weeks at −80°C or 37°C. Data from a representative experiment that was performed twice are shown. (D) Guanidine denaturation of trimeric DS-Cav1, trimeric DS-Cav1-I53-50A, and DS-Cav1-I53-50 nanoparticles. Two related measures of intrinsic fluorescence are plotted: barycentric mean (left) and the position of the emission peak (right). The native state of the DS-Cav1-I53-50 nanoparticles is red-shifted relative to DS-Cav1 and trimeric DS-Cav1-I53-50 due to the presence of a solvent-exposed tryptophan in I53-50B.4PT1. Circles represent the arithmetic mean and error bars the standard deviation of measurements from three independently prepared samples. (E) Summary of HDX-MS comparison of trimeric DS-Cav1 and DS-Cav1-I53-50 nanoparticles. Each observed peptide was classified as more (green) or less (orange) stable in DS-Cav1-I53-50 (or unchanged; yellow). Portions of the structure for which peptides were not observed in both datasets are gray. See also Figure S4, Figure S5, Figure S6 and Table S1.

**Figure S4:**
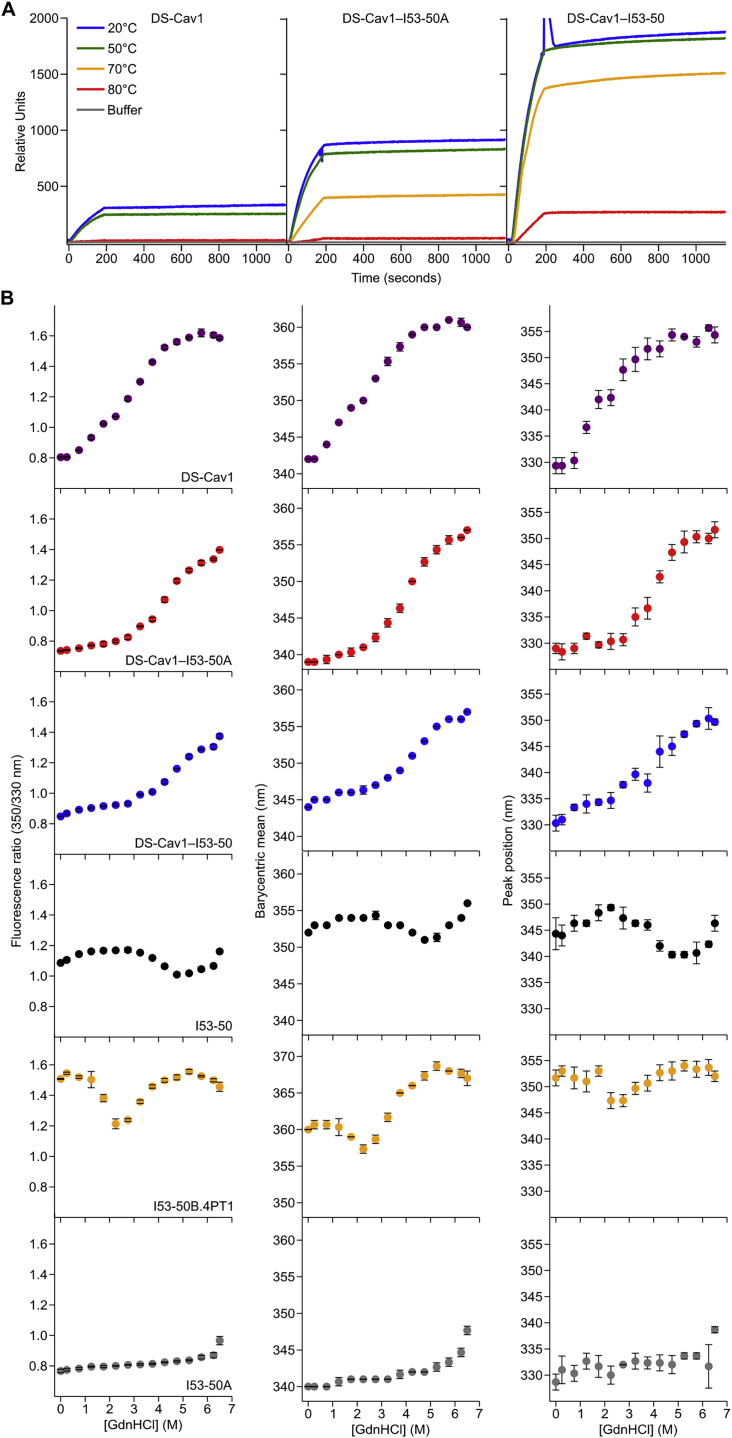
Characterization of Physical Stability by D25 Binding and Chemical Denaturation, Related to Figure 2 (A) Raw SPR data for the experiment presented in Figure 2A (retention of D25 binding after thermal stress). Representative data are shown from an experiment that was performed twice. (B) Guanidine denaturation of DS-Cav1, trimeric DS-Cav1–I53-50A, DS-Cav1–I53-50 nanoparticles, trimeric I53-50A, pentameric I53-50B.4PT1 and unmodified I53-50 nanoparticles monitored by intrinsic fluorescence. The fluorescence ratio (350/330), barycentric mean, and peak position are plotted as in Figure 2D. Dots represent the arithmetic mean and error bars the standard deviation of measurements from three independently prepared samples.

Given the key antigenic properties of prefusion F, we used three orthogonal approaches to measure the physical stability of DS-Cav1 when fused to I53-50A and when further assembled into the icosahedral nanoparticle. The first assay measured the retention of prefusion-specific mAb binding after thermal stress (Joyce et al., 2016, Krarup et al., 2015, McLellan et al., 2013b). We found that trimeric DS-Cav1, trimeric DS-Cav1-I53-50A, and DS-Cav1-I53-50 nanoparticles all bound D25 equivalently after incubation for 1 h at 20°C and 50°C, but lost most of their reactivity after 1 h at 80°C (Figure 2A). Interestingly, while D25 was also unable to bind trimeric DS-Cav1 incubated at 70°C for 1 h, trimeric DS-Cav1-I53-50A and the DS-Cav1-I53-50 nanoparticles retained 50% and 80% of their respective binding signals (Figures 2A and S4A). While the multivalent nature of the DS-Cav1-I53-50 nanoparticles complicates direct quantitative comparisons to trimeric DS-Cav1, these results indicate that genetic fusion to the I53-50A trimer further stabilizes the prefusion conformation of DS-Cav1 and suggest that this increased stability is maintained in the context of the assembled nanoparticle immunogen. AM14 binding measured by bio-layer interferometry and SEC further showed that samples of DS-Cav1-I53-50A stored for 1 and 2 weeks at 37°C revealed no discernible loss in antigenicity relative to samples stored at −80°C and exhibited no signs of protein degradation or aggregation (Figures 2B and 2C).

We used chemical denaturation in guanidine hydrochloride (GdnHCl) monitored by intrinsic fluorescence as a second, antibody-independent technique to evaluate physical stability. Analyzing fluorescence emission from DS-Cav1 incubated in 0–6.5 M GdnHCl revealed that the protein undergoes two subtly distinct transitions, one between 0.25 and 2.25 M GdnHCl and another between 2.25 and 5.75 M (Figures 2D and S4B). In contrast, only a single transition between 2.25 and 6.25 M GdnHCl is apparent for trimeric DS-Cav1-I53-50A, further supporting that the native conformation of DS-Cav1 is stabilized by genetic fusion to trimeric I53-50A. Comparing the data for the DS-Cav1-I53-50 nanoparticle and unmodified I53-50 (lacking fused DS-Cav1) indicated that the stabilization is maintained upon assembly to the icosahedral nanoparticle (Figures 2D and S4B). The source of this effect is likely the extreme stability of the I53-50A trimer, which only began to exhibit small changes in fluorescence at very high (≥5.75 M) GdnHCl concentrations (Figure S4B).

**Figure S5:**
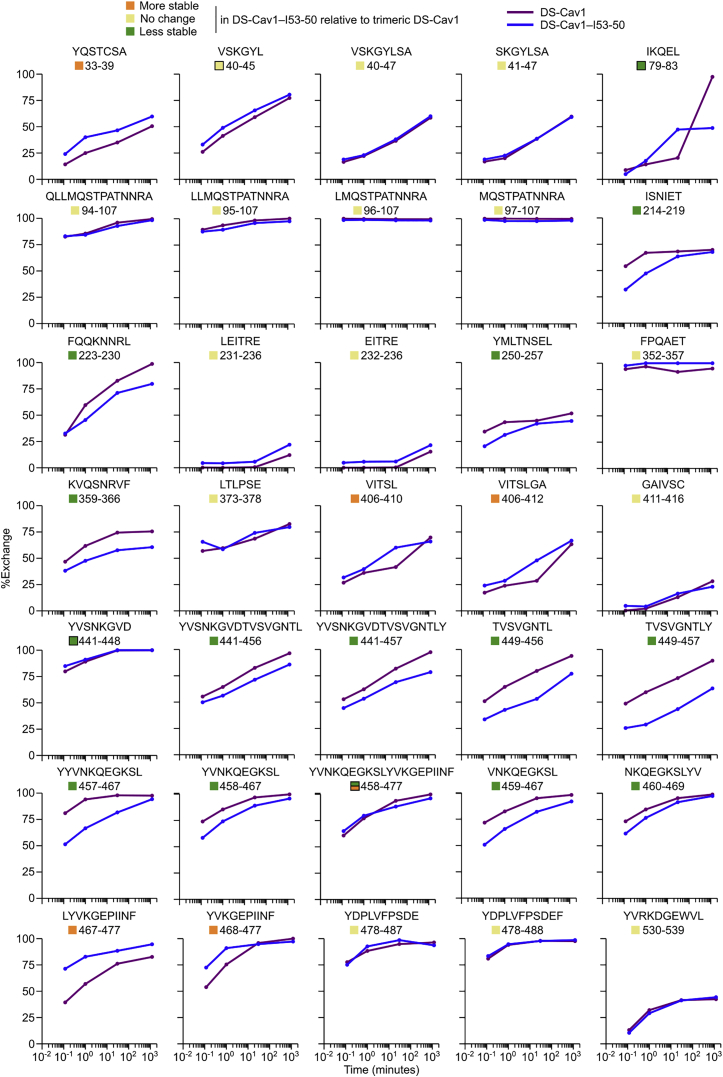
Individual HDX Plots for DS-Cav1 Peptides Derived from Trimeric DS-Cav1 and DS-Cav1–I53-50, Related to Figure 2 Percent exchange relative to a totally deuterated control is plotted for each peptide that was observed in both trimeric DS-Cav1 (purple) and DS-Cav1–I53-50 (blue). Each point represents the average from duplicate measurements. Only peptides that displayed unimodal exchange behavior are included here; those that exhibited bimodal behavior are presented in Figure S6. Squares to the left of the peptide position indicate whether the peptide is more stable, less stable, or unchanged in DS-Cav1–I53-50 compared to trimeric DS-Cav1 and matches the coloring of that peptide in the structure presented in Figure 2C. Certain regions were covered by multiple peptides, one of which was used to determine whether that region was more stable, less stable, or unchanged. The color squares for peptides that were not used include a black outline, and the corresponding peptides selected to indicate comparative behavior are as follows: VSKGYL 40-45, VSKGYLSA 40-47; IQKEL 79-83, IKQELDKYKNAVTEL 79-93 (bimodal data in Figure S6); YVSNKGVD 441-448, YVSNKGVDTVSVGNTL 441-456 and YVSNKGVDTVSVGNTLY 441-457; YVNKQEGKSLYVKGEPIINF 458-477, VNKQEGKSL 459-467 and NKQEGKSLYV 460-469 (first half; more stable); YVNKQEGKSLYVKGEPIINF 458-477, LYVKGEPIINF 467-477 and YVKGEPIINF 468-477 (second half; less stable).

**Figure S6:**
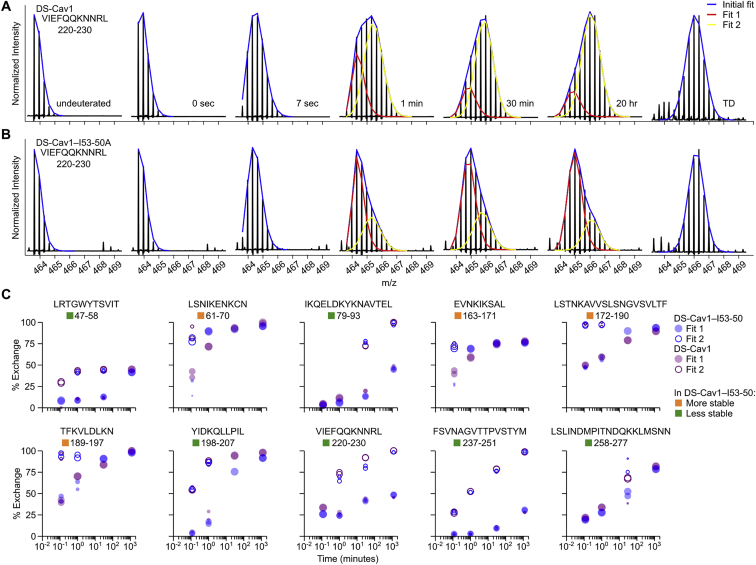
Example of Bimodal Deconvolution of HDX-MS Spectra and HDX Plots for Peptides Displaying Bimodal Deuteration Profiles, Related to Figure 2 (A) Raw spectra for certain peptides displayed spectral broadening and were better fit by bimodal deconvolution (Guttman et al., 2013, Weis et al., 2006). Example spectra corresponding to peptide 220-VIEFQQKNNRL-230 in trimeric DS-Cav1 are shown. Fit 1 appears to represent a slower-exchanging population of molecules, whereas Fit 2 appears to represent a faster-exchanging population. The total incubation time in D_2_O is indicated next to each spectrum. TD, totally deuterated control. (B) Example of spectra from the same peptide in DS-Cav1–I53-50 that also showed bimodal exchange kinetics. In this example, a higher fraction of the population of molecules is fit by Fit 1 for DS-Cav1–I53-50 relative to trimeric DS-Cav1, suggesting less dynamic behavior when presented on the nanoparticle. (C) “Bubble plots” that represent deuteration of peptides that exhibited bimodal HDX kinetics. Purple dots represent DS-Cav1 trimer and blue dots represent DS-Cav1–I53-50 nanoparticle. Filled and open circles are used for fits 1 (slower exchange) and 2 (faster exchange), respectively, and the area of each circle is proportional to the population fraction accounted for by its corresponding fit. Each data point from duplicate measurements of each peptide is shown. For spectra that could be adequately fit by a single distribution, a filled circle is used. Squares to the left of the peptide position indicate whether the peptide is more stable, less stable, or unchanged in DS-Cav1–I53-50 compared to trimeric DS-Cav1 and matches the coloring of that peptide in the structure presented in Figure 2C.

Finally, we used hydrogen-deuterium exchange mass spectrometry (HDX-MS) to probe the local structural stability of DS-Cav1 as a soluble trimer and in the context of the assembled DS-Cav1-I53-50 nanoparticle. HDX-MS revealed an overall increase in local stability in the context of the DS-Cav1-I53-50 nanoparticle, including in key antigenic sites for neutralizing antibodies such as site Ø and site II (Figures 2E, S5, and S6). Several epitopes of interest, including site Ø and site II, displayed bimodal HDX profiles with DS-Cav1 generally exhibiting a substantial bias toward the more ordered conformational states when presented on the I53-50 nanoparticle (Figure S6). Antigenic site V was an exception to the stabilizing trend, as this site displayed a bias toward a more dynamic conformation in DS-Cav1-I53-50 compared to trimeric DS-Cav1.

### Preparation and Formulation of Nanoparticle Immunogens

**Figure 3:**
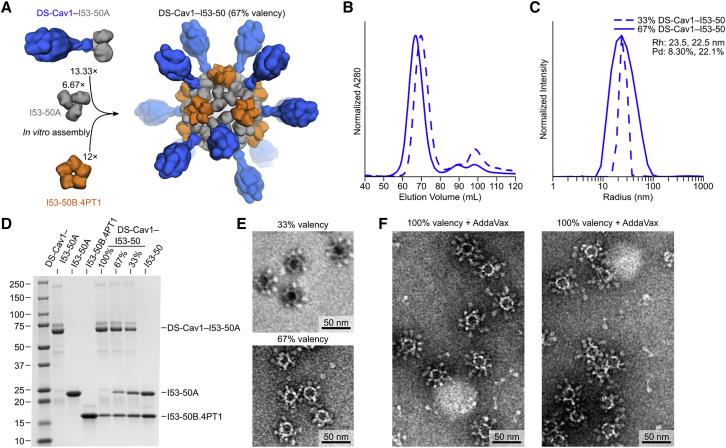
Preparation and Formulation of Nanoparticle Immunogens (A) Schematic representation of *in vitro* assembly of partial valency nanoparticle immunogens. Although a single structural model is shown for clarity, the geometric distribution of antigen on the nanoparticle exteriors is expected to be random due to the icosahedral symmetry of I53-50. (B) SEC chromatograms of 33% and 67% DS-Cav1-I53-50 nanoparticles, showing efficient assembly to the icosahedral state (60–80 mL) with small amounts of residual components (85–105 mL). (C) Dynamic light scattering of 33% and 67% DS-Cav1-I53-50 nanoparticles. The hydrodynamic radius (Rh) and polydispersity (Pd) of each nanoparticle are indicated. (D) Reducing SDS-PAGE of SEC-purified components and nanoparticle immunogens. Molecular weight marker is indicated in kilodaltons. (E) Negative stain electron micrographs of 33% and 67% valency DS-Cav1-I53-50 nanoparticle immunogens. (F) Negative stain electron microscopy of 100% valency DS-Cav1-I53-50 nanoparticles formulated in AddaVax. See also Table S1.

For immunogenicity studies, we exploited the two-component nature of the I53-50 scaffold to produce a series of nanoparticle immunogens displaying DS-Cav1 at various densities. Immunogens bearing DS-Cav1 in only 33% or 67% of the 20 available locations were prepared by simply mixing DS-Cav1-bearing and unmodified trimeric I53-50A at 1:2 or 2:1 molar ratios, respectively, prior to addition of the pentameric component to drive nanoparticle assembly (Figure 3A). Assembly to the same icosahedral architecture as 100% valency DS-Cav1-I53-50 was confirmed by DLS and EM after SEC-based purification, while SDS-PAGE enabled visualization of the ratio of DS-Cav1-bearing to unmodified trimeric I53-50A in the SEC-purified nanoparticle immunogens (Figures 3B–3E). Negative stain electron micrographs of 100% valency DS-Cav1-I53-50 nanoparticles formulated in AddaVax, a squalene-based oil-in-water emulsion, showed fields of monodisperse particles with visible displayed antigen as well as pale lipid droplets (Figure 3F), indicating that the adjuvant had no discernible adverse effects on the structure of the nanoparticle immunogen.

### Multivalent Presentation on I53-50 Enhances DS-Cav1 Immunogenicity in Mice

**Figure 4:**
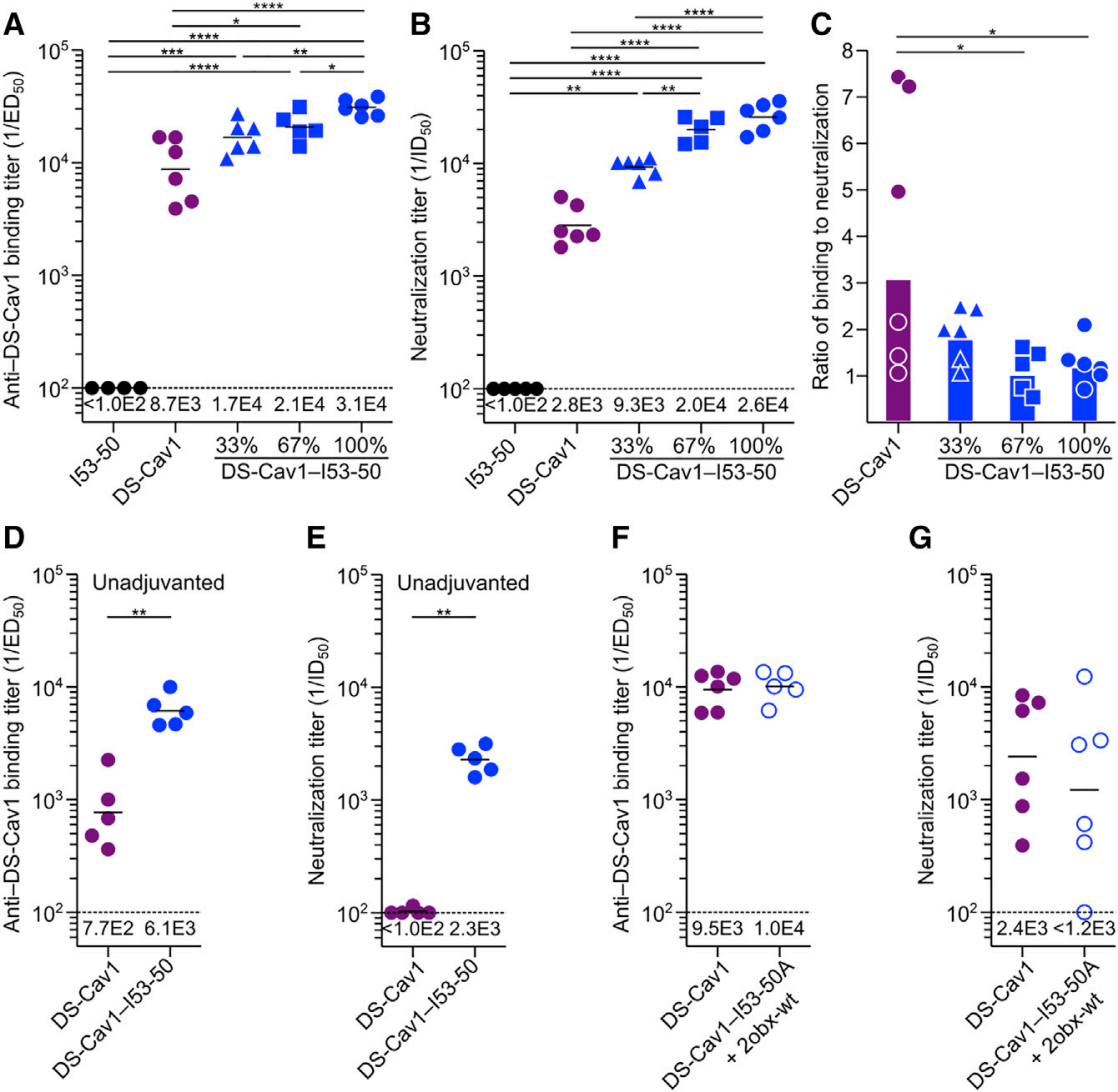
Multivalent Presentation on I53-50 Enhances DS-Cav1 Immunogenicity in Mice (A) DS-Cav1-specific binding antibody titers from mice immunized with I53-50, trimeric DS-Cav1, or DS-Cav1-I53-50 nanoparticle immunogens at the indicated valency. Each symbol represents serum from an individual animal, and the geometric mean for each group is indicated by a horizontal line and provided at the bottom of the plot. The dotted line represents the lower limit of detection for the assay. (B) Serum neutralizing antibody titers induced by each immunogen, plotted as in (A). (C) Ratio of DS-Cav1-binding to neutralizing antibody titers, derived from the data in (A) and (B). Each symbol represents the ratio of ED_50_:ID_50_ for the sera from an individual animal; the bars represent the geometric mean for each group. (D) DS-Cav1-specific binding antibody titers from mice immunized with trimeric DS-Cav1 or DS-Cav1-I53-50 nanoparticles without adjuvant, plotted as in (A). (E) Serum neutralizing antibody titers induced by each immunogen without adjuvant, plotted as in (B). (F) DS-Cav1-specific binding antibody titers from mice immunized with trimeric DS-Cav1 or a non-assembling mixture of trimeric DS-Cav1-I53-50A and 2obx-wt. ELISA data plotted as in (A). (G) Serum neutralizing antibody titers induced by DS-Cav1 or a non-assembling mixture of trimeric DS-Cav1-I53-50A and 2obx-wt, plotted as in (B). The ELISA and neutralization data shown are from representative experiments that were each performed at least twice. Statistical significance was calculated using the two-tailed non-parametric Mann-Whitney U test for two groups’ comparison or one-way ANOVA with multiple comparisons corrected by Tukey’s test when three or more groups were compared. ^∗^p < 0.05; ^∗∗^p < 0.01; ^∗∗∗^p < 0.001; ^∗∗∗∗^p < 0.0001. See also Table S1.

We next compared the immunogenicity of trimeric DS-Cav1 to the I53-50 nanoparticles displaying DS-Cav1 at various densities in BALB/c mice. All immunogens were formulated in AddaVax and each injection comprised 5 μg of DS-Cav1 antigen (or 5 μg unmodified I53-50). As expected, immunization with unmodified I53-50 nanoparticles induced no detectable response against DS-Cav1, while sera from animals immunized with trimeric DS-Cav1 contained antigen-specific antibodies and neutralized virus (Figures 4A and 4B). All three of the nanoparticle immunogens induced more robust humoral responses than trimeric DS-Cav1 and revealed a correlation between antigen density on the nanoparticle exterior and the magnitude of the response. At 100% valency, DS-Cav1-I53-50 induced 3-fold higher antigen-specific antibody titers than trimeric DS-Cav1 and 9-fold higher neutralizing antibody titers (calculated using the geometric mean of each group). The decreased ratio of binding to neutralizing antibodies elicited by DS-Cav1-I53-50 compared to trimeric DS-Cav1 (1.2:1 versus 3.1:1) suggests that the quality of the antibody response is improved by displaying DS-Cav1 on the I53-50 scaffold (Figure 4C). One potential explanation for this improvement is that potent neutralizing epitopes, such as site Ø, are more exposed on the nanoparticle and are therefore more accessible to BCRs than less neutralizing or non-physiological epitopes like the foldon. Immunization of mice with trimeric DS-Cav1 or DS-Cav1-I53-50 in the absence of adjuvant led to similar results. We observed low binding and neutralizing titers for unadjuvanted trimeric DS-Cav1, while unadjuvanted DS-Cav1-I53-50 elicited binding and neutralization titers similar to AddaVax-formulated trimeric DS-Cav1 (Figures 4D and 4E). These data indicate that the designed nanoparticle immunogens are substantially more immunogenic than trimeric DS-Cav1 and support the hypothesis that the increased immunogenicity derives, at least in part, from efficient BCR cross-linking by the dense array of antigen on the nanoparticle surface.

To better understand the basis for the enhanced immunogenicity of DS-Cav1-I53-50, we compared the antibody response induced by trimeric DS-Cav1 to a mixture of trimeric DS-Cav1-I53-50A and 2obx-wt, a variant of the I53-50B pentamer lacking the computationally designed protein-protein interface that drives nanoparticle assembly. We observed no significant difference in the levels of DS-Cav1-specific antibodies or neutralizing antibodies (Figures 4F and 4G), demonstrating that the enhanced immunogenicity of the DS-Cav1-I53-50 nanoparticle does not derive from the physical stabilization afforded by genetic fusion to I53-50A or from T cell epitopes within the I53-50A subunit. Instead, the data directly link enhanced immunogenicity to formation of the icosahedral nanoparticle.

### Antibody Response to the I53-50 Nanoparticle Scaffold

**Figure 5:**
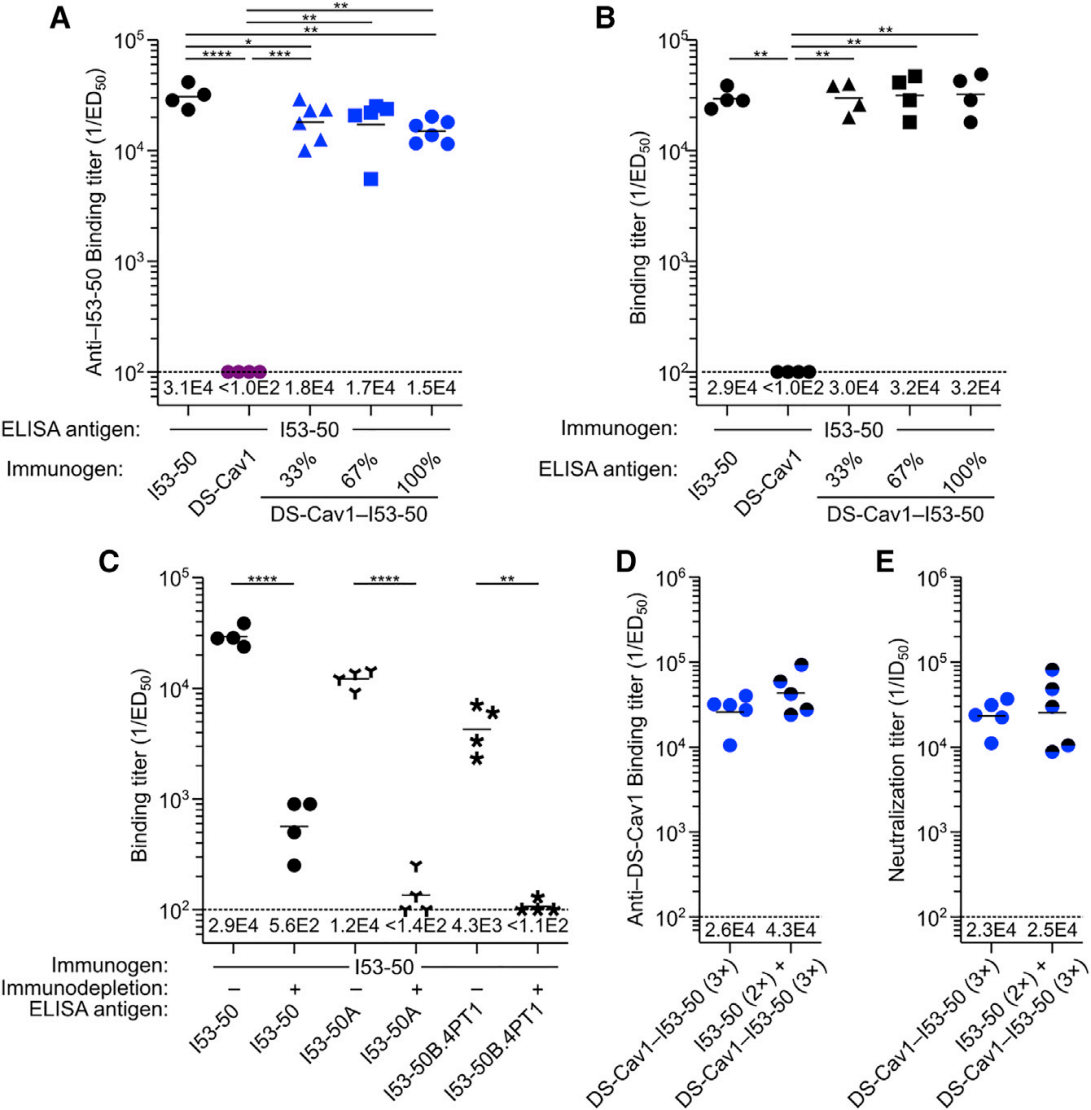
Antibody Response to the I53-50 Nanoparticle Scaffold (A) I53-50-specific binding antibody titers, plotted as in Figure 4A. The sera from all groups were analyzed for binding to unmodified I53-50 nanoparticles. (B) Sera from mice immunized with unmodified I53-50 nanoparticles were analyzed for binding antibody titer against several antigens, plotted as in Figure 4A. The first groups in (A) and (B) are technical replicates. (C) Sera from mice immunized with unmodified I53-50 nanoparticles were analyzed for antibody titer against I53-50 nanoparticles (same data as the first group in B), trimeric I53-50A, and pentameric I53-50B.4PT1 before or after immunodepletion with I53-50, plotted as in Figure 4A. (D) DS-Cav1-specific binding antibody titers from mice immunized three times with DS-Cav1-I53-50 with and without pre-immunization with unmodified I53-50, plotted as in Figure 4A. (E) Serum neutralizing antibody titers corresponding to the immunizations in (D), plotted as in Figure 4B. Statistical significance was calculated using the two-tailed non-parametric Mann-Whitney U test for two groups’ comparison or one-way ANOVA with multiple comparisons corrected by Tukey’s test when three or more groups were compared. ^∗^p < 0.05; ^∗∗^p < 0.01; ^∗∗∗^p < 0.001; ^∗∗∗∗^p < 0.0001. See also Table S1.

In addition to evaluating the anti-DS-Cav1 response, we measured the induction of I53-50-specific antibodies. A robust antibody response was elicited by unmodified I53-50 nanoparticles, whereas the responses were lower when DS-Cav1 was displayed on the nanoparticle exterior at any valency (Figure 5A). To determine whether the lower anti-I53-50 responses were due to physical shielding (Collins et al., 2017), we used sera from the animals immunized with unmodified I53-50 to analyze antibody binding to the nanoparticles displaying DS-Cav1. We observed no difference between serum antibody binding to unmodified I53-50 and DS-Cav1-I53-50 nanoparticles displaying the antigen at any valency (Figure 5B). These data indicate that DS-Cav1 does not sterically prevent antibody access to the I53-50 nanoparticle surface even when genetically fused to the N terminus of all 20 trimeric components, a result that is consistent with the spacing of DS-Cav1 on the particle surface (Figure 1B) and the long, flexible linker connecting them.

To further dissect the anti-scaffold response, we measured antibody binding to each component of I53-50 before and after immunodepleting sera using assembled, unmodified I53-50 nanoparticles. In this experiment, immunodepletion eliminates antibodies directed against the nanoparticle exterior, and residual antibody binding to the components measures responses against epitopes on the interior surface of the nanoparticle or buried upon nanoparticle assembly. As expected, prior to immunodepletion, robust levels of antibodies specific to the assembled I53-50 nanoparticles, the I53-50A trimer, and the I53-50B.4PT1 pentamer were observed in sera from animals immunized with unmodified I53-50 (Figure 5C). Immunodepletion reduced antibody titers against assembled I53-50 nanoparticles and the individual components by 98%–99%. Near-total depletion of component-specific antibody by adsorption to assembled I53-50 nanoparticles suggests that the nanoparticle immunogens do not present their interior surfaces to B cells to an appreciable extent.

To determine whether pre-existing immunity against the nanoparticle scaffold deleteriously affects the performance of DS-Cav1-I53-50 as an immunogen, we pre-immunized mice with unmodified I53-50 to induce anti-scaffold antibodies and compared DS-Cav1-specific and neutralizing antibody titers to a control group that did not receive the pre-immunizations. Both the mean binding and neutralizing titers of the pre-immunized group were slightly higher than the control group, although the differences were not significant (Figures 5D and 5E). This result demonstrates that the presence of anti-scaffold antibodies does not adversely affect the potent antigen-specific response induced by DS-Cav1-I53-50.

### Cellular Responses to I53-50, DS-Cav1, and DS-Cav1-I53-50

**Figure 6:**
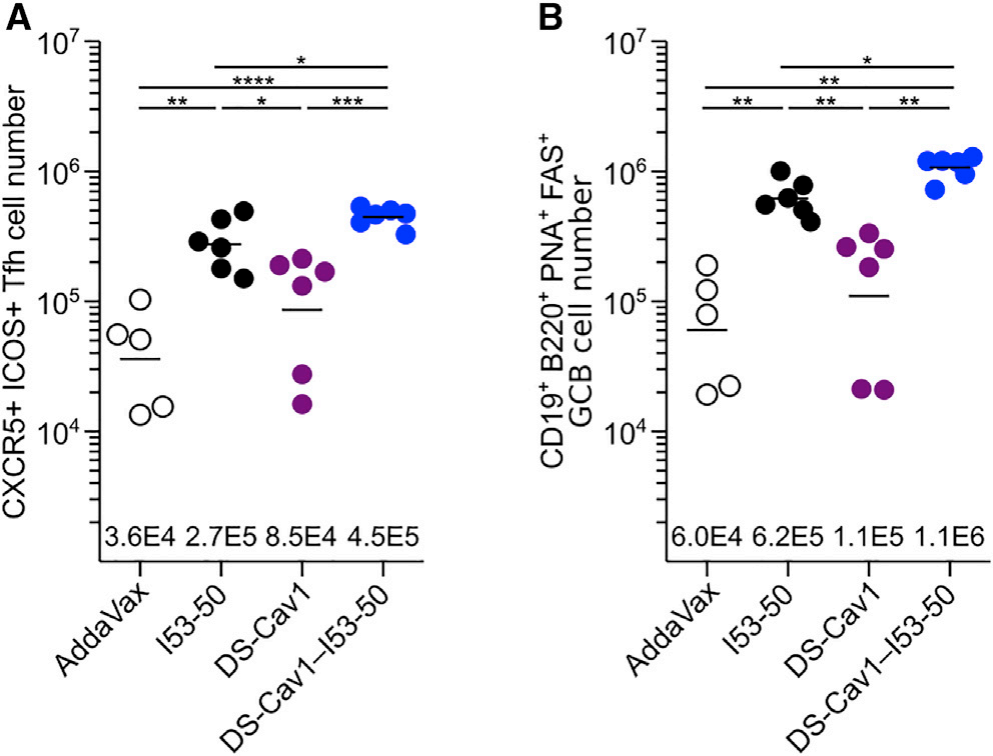
Cellular Responses to I53-50, Trimeric DS-Cav1, and DS-Cav1-I53-50 (A) Quantitation of total Tfh cells from draining lymph node cell suspensions. Each symbol represents a measurement from an individual mouse and the geometric mean for each group is indicated by a horizontal line and provided at the bottom of the plot. The data shown are from a representative experiment that was performed twice with groups of 6 to 7 mice. (B) Quantitation of total GC B cells from draining lymph node cell suspensions, plotted as in (A). Statistical significance was calculated using one-way ANOVA with multiple comparisons corrected by Tukey’s test. ^∗^p < 0.05; ^∗∗^p < 0.01; ^∗∗∗^p < 0.001; ^∗∗∗∗^p < 0.0001. See also Table S1.

To determine whether T follicular helper (Tfh) cells, essential regulators of B cell maturation in germinal centers (GCs) (Crotty, 2014), play a role in the enhanced immunogenicity of DS-Cav1-I53-50, we compared the total numbers of Tfh and GC B cells induced by each immunogen. We observed a 5.3-fold increase in the number of total Tfh cells in mice immunized with DS-Cav1-I53-50 versus trimeric DS-Cav1 (Figure 6A). The particulate nature of DS-Cav1-I53-50 appears to play a key role in this increase, as unmodified I53-50 nanoparticles also induced high numbers of Tfh cells. This trend was slightly more pronounced in the numbers of total GC B cells induced by each immunogen (Figure 6B). These data are consistent with previous studies showing that particulate immunogens enhance GC formation and Tfh expansion compared to soluble antigen (Moon et al., 2012).

### Immunogenicity of DS-Cav1-I53-50 in Nonhuman Primates

**Figure 7:**
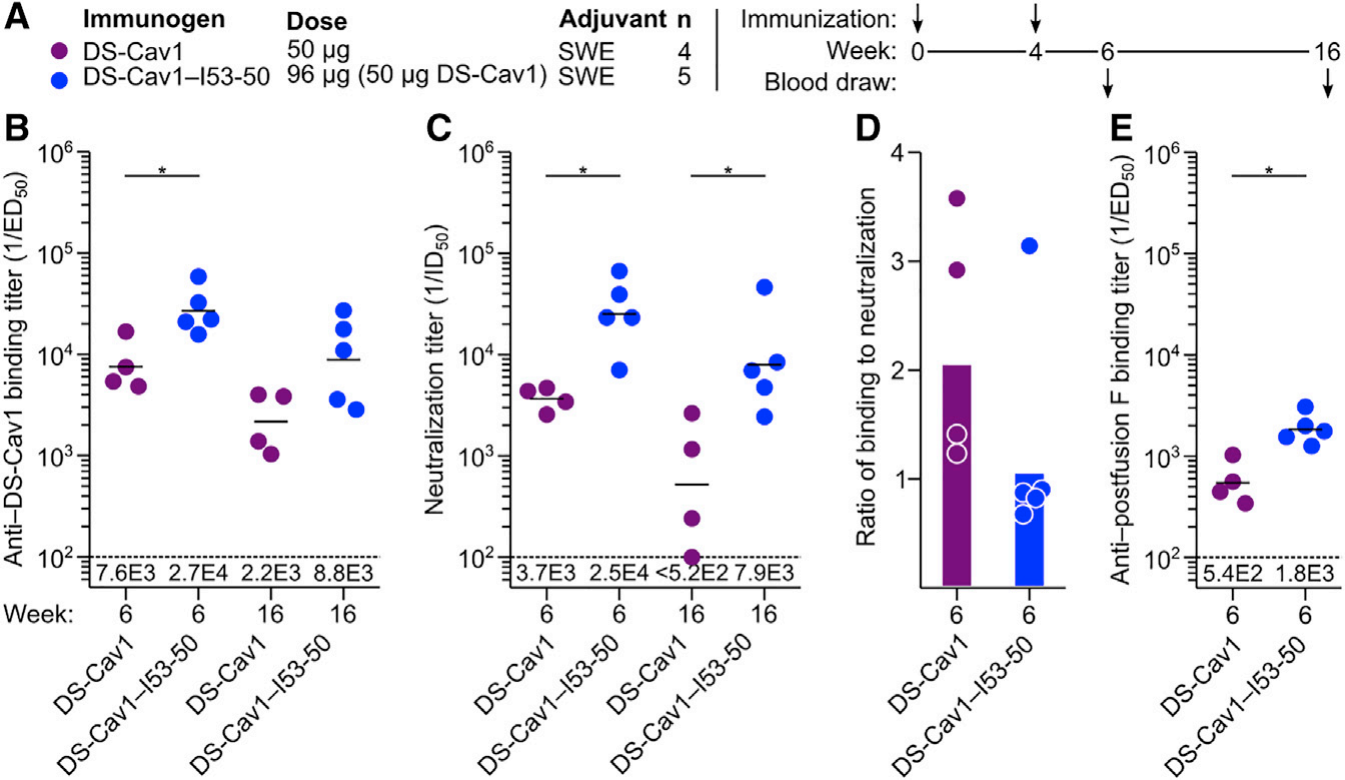
Immunogenicity of DS-Cav1-I53-50 in Nonhuman Primates (A) Study design. (B) DS-Cav1-specific binding antibody titers, plotted as in Figure 4A. (C) Serum neutralizing antibody titers, plotted as in (B). (D) Ratio of DS-Cav1-binding to neutralizing antibody titers, plotted as in Figure 4C. (E) Postfusion F-specific antibody titers after immunodepletion with trimeric DS-Cav1, plotted as in Figure 4A. Statistical significance was calculated using the two-tailed non-parametric Mann-Whitney U test for two groups’ comparison. ^∗^p < 0.05. See also Figure S7 and Table S1.

**Figure S7:**
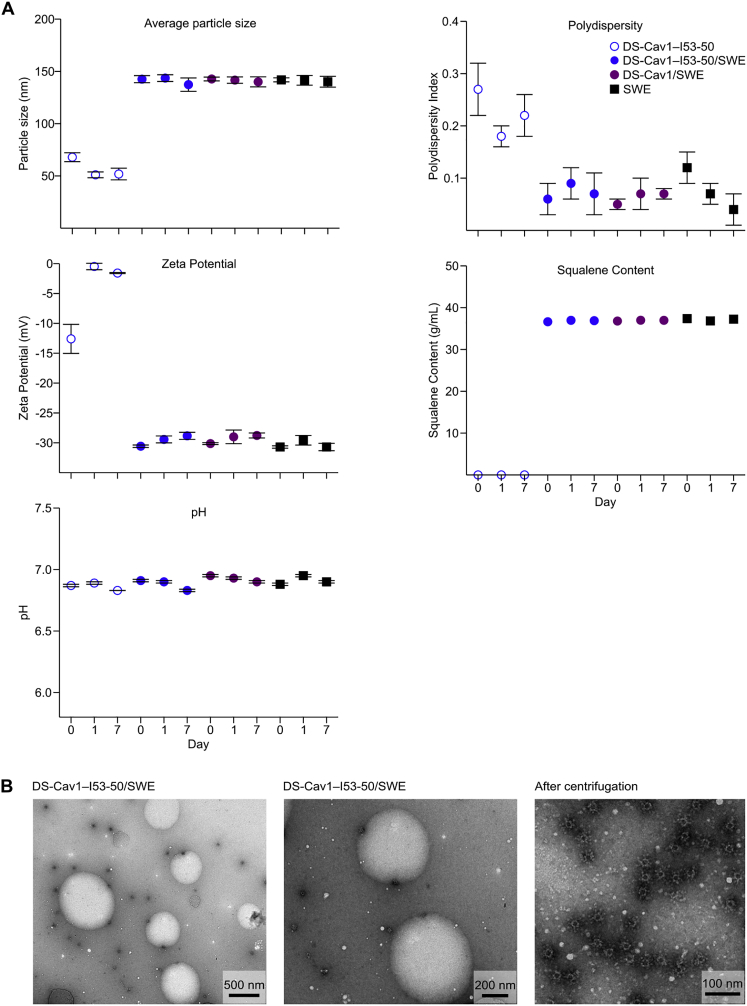
Physicochemical Characterization of Formulations of DS-Cav1 and DS-Cav1-I53-50 with SWE Adjuvant, Related to Figure 7 (A) Trimeric DS-Cav1 and DS-Cav1–I53-50 were formulated with SWE adjuvant and characterized at day 0 and after incubation at 4°C for 1 day and 7 days. DS-Cav1–I53-50 alone and SWE alone were included as controls. Graphs show particle size, polydispersity index, zeta potential, squalene content, and pH. Physicochemical parameters of both the DS-Cav1 and DS-Cav1–I53-50 formulations were stable in the presence of SWE. (B) Negative stain EM analysis of DS-Cav1–I53-50 in SWE adjuvant was performed after incubation at 4°C for 2 weeks. Pictures show nanoparticles and SWE droplets at different magnifications before centrifugation (left and middle panel) and nanoparticles after ultracentrifugation (right panel). Intact nanoparticles are clearly visible after the 2-week incubation with SWE.

To determine whether the enhanced immunogenicity we observed in mice is also obtained in a species closer to humans, we immunized Indian rhesus macaques with trimeric DS-Cav1 or DS-Cav1-I53-50 formulated in SWE, a squalene-based oil-in-water emulsion (Ventura et al., 2013) (Figures 7A and S7). In agreement with the data obtained in mice, DS-Cav1-I53-50 induced higher antigen-specific (Figure 7B) and neutralizing (Figure 7C) antibody titers than trimeric DS-Cav1, and the ratio of binding to neutralizing antibodies was again lower for the nanoparticle immunogen (Figure 7D). Immunodepletion of the week 6 sera with trimeric DS-Cav1 followed by measurement of residual antibody binding to postfusion F (Corti et al., 2013) showed that, for both immunogens, roughly 90% of the F-specific antibodies are directed at epitopes in prefusion F (Figure 7E). Together, these data confirm in a primate immune system the enhanced immunogenicity conferred by multivalent presentation of DS-Cav1 on I53-50 and are consistent with the formulated nanoparticle immunogen maintaining DS-Cav1 in the prefusion conformation *in vivo*.

## Discussion

We have shown that computationally designed two-component protein nanomaterials are capable of scaffolding and stabilizing DS-Cav1, a complex viral glycoprotein antigen, and that I53-50-based immunogens induce potent RSV-neutralizing antibody responses. The ability to design new self-assembling protein complexes with atomic-level accuracy enables the production of materials with structural features tailored to specific applications (Bale et al., 2016, Hsia et al., 2016, King et al., 2012, King et al., 2014). In this work, we have exploited this capability at the level of both the oligomeric building block as well as the assembled nanomaterial. At the building block level, we genetically fused trimeric DS-Cav1 to a variety of designed nanoparticle components with matching 3-fold symmetry and exterior-facing N termini. At the assembled nanomaterial level, we selected for immunogenicity studies the self-assembling scaffold that maximized antigen density. These and other features can be further explored and optimized in future efforts, and we anticipate that by enabling precise and systematic variation of structural parameters such as overall immunogen size or spacing between antigens, computationally designed self-assembling immunogens could enable better definition of the structural correlates of immunogenicity.

The construction of two-component nanomaterials like I53-50 from multiple copies of two distinct protein subunits distinguishes them from homomeric self-assembling protein scaffolds such as ferritin. In this work, we took advantage of the two-component nature of I53-50 in several ways. First, analysis of the isolated components enabled more detailed and facile characterization of the stability of each protein in the system, revealing that it is genetic fusion of DS-Cav1 to the I53-50A component—not nanoparticle assembly—that results in stabilization of the prefusion conformation beyond that afforded by the DS-Cav1 mutations. Second, we exploited the control afforded by *in vitro* assembly to tune the density of antigen on the nanoparticle exterior by simply assembling mixtures of trimeric components containing or lacking fused DS-Cav1 at defined ratios. The correlation we observed between increased antigen density and immunogenicity agrees with previous work using other multivalent antigen presentation platforms (for review, see Frietze et al., 2016, López-Sagaseta et al., 2015) and supports the notion that efficient cross-linking of BCRs by high-density antigen arrays is a key driver of immunogenicity (Abbott et al., 2018, Bachmann and Jennings, 2010). Third, our ability to easily prepare an immunogen nearly identical to DS-Cav1-I53-50 in terms of protein content that nevertheless is unable to assemble enabled the observation that the increase in antigen-specific antibodies induced by DS-Cav1-I53-50 is directly linked to formation of the nanoparticle structure. These observations motivate the design of additional self-assembling scaffolds that enable antigen display at still higher valency, as well as systematic investigation of the roles of antigen flexibility, antigen-antigen spacing, and linker design in the performance of nanoparticle immunogens.

The recent generation of stabilized prefusion F antigens has breathed new life into RSV vaccine development (Graham, 2016, Joyce et al., 2016, Krarup et al., 2015, McLellan et al., 2013b). Although there is no formal correlate of protection from infection, several lines of evidence indicate that high levels of neutralizing antibodies protect against severe RSV disease (Falsey and Walsh, 1998, Glezen et al., 1986, Piedra et al., 2003). We found that in RSV-naive mice and nonhuman primates DS-Cav1-I53-50 induces roughly 10-fold higher levels of neutralizing antibodies than trimeric DS-Cav1, a leading clinical-stage RSV vaccine candidate. It is possible that second-generation prefusion RSV F antigens with improved stability and immunogenicity (Joyce et al., 2016) would yield even more potent responses when presented in multivalent form on I53-50 or other nanoparticles. We note that one limitation of RSV-naive animal models is that prior RSV infection is universal among adult humans, and a vaccine will presumably work in part by boosting pre-existing immunity (Graham, 2016). RSV-primed animal models (Blanco et al., 2018, Steff et al., 2017) could be useful for simulating this scenario and evaluating DS-Cav1-I53-50 in a more clinically relevant context.

Anti-scaffold antibody responses are typically observed when heterologous antigens are presented on self-assembling protein scaffolds, which raises three main questions. First, will the scaffold generate cross-reactive responses against human (self) proteins? The two subunits of I53-50 are both derived from bacterial enzymes that have no detectable homology to any human proteins, making the induction of cross-reactive antibodies unlikely, although this would need to be closely monitored in any clinical-stage program. Second, do anti-scaffold responses detract from or compete with the antigen-specific response? Our data are in accordance with previous studies (Aide et al., 2011, Kanekiyo et al., 2013) in that presentation of DS-Cav1 on the I53-50 nanoparticle induced higher antigen-specific responses despite the presence of an anti-scaffold response, including after multiple boosts. Third, would anti-scaffold antibodies prevent use of the same scaffold in prime-boost regimens or in multiple vaccines? We found that pre-existing immunity against the I53-50 scaffold did not deleteriously affect the antigen-specific response, similar to observations in clinical studies of RTS,S, a recombinant nanoparticle vaccine for malaria (Aide et al., 2011). However, to fully understand the role of anti-scaffold responses, additional studies, ideally in humans, will be required.

In conclusion, we have described a versatile two-component protein nanoparticle platform for multivalent presentation of complex antigens. Given the rapidly expanding capabilities of computational protein design (Huang et al., 2016), continued development of this structure-based approach to designing self-assembling immunogens could be useful for improving the potency, durability, and breadth of vaccines against a number of important pathogens.

## STAR★Methods

### Key Resources Table

**Table:** 

REAGENT or RESOURCE	SOURCE	IDENTIFIER
**Antibodies**		

Mouse CD4-PO (clone RM4-5)	Thermo Fisher Scientific	Cat #14-0042-82; RRID: AB_467067
Mouse CD3-PE-Cy7 (clone 17A2)	BioLegend	Cat #100220; RRID: AB_1732057
Mouse CD45.1-PB (clone A20)	BioLegend	Cat #110722; RRID: AB_492866
Mouse PD1-FITC (clone RMP1-30)	Thermo Fisher Scientific	Cat #11-9981-81; RRID: AB_465466
Mouse ICOS-PE (clone 7E.17G9)	BioLegend	Cat #117406; RRID: AB_2122712
Mouse CXCR5-biotin (clone 2G8)	BD Biosciences	Cat #551960; RRID: AB_394301
Mouse B220-PO (clone RA3-6B2)	Thermo Fisher Scientific	Cat #RM2630; RRID: AB_10372219
Mouse CD19-PE-Cy7 (clone 1D3)	BD Biosciences	Cat #552854; RRID: AB_394495
Mouse FAS-PE (clone Jo2)	BD Biosciences	Cat #554258; RRID: AB_395330
Mouse PNA-Fluorescein	Vector Laboratories	Cat #FL-1071; RRID: AB_2315097
Streptavidin-APC	Thermo Fisher Scientific	Cat #SA1005
Human CD4-PE-TexasRed (clone S3.5)	Thermo Fisher Scientific	Cat #MHCD0417; RRID: AB_10371766
Human CD45RA-QD655 (clone MEM-56)	Beckman Coulter	Cat #IM1834U
Human CCR7-BV421 (clone G043H7)	BioLegend	Cat #353208; RRID: AB_11203894
Human CD14- PE-Cy5 (clone RMO52)	Beckman Coulter	Cat #A07765
Human CD25-PE-Cy5 (clone B1.49.9)	Beckman Coulter	Cat #IM0478U; RRID: AB_130985
Human CD8- PE-Cy5 (clone B9.11)	Beckman Coulter	Cat #A07756; RRID: AB_1575981
Human CD16- PE-Cy5 (clone 3G8)	Beckman Coulter	Cat #A07767
Human CD19- PE-Cy5 (clone J3-119)	Beckman Coulter	Cat #A07771
Human CD56- PE-Cy5 (clone N901)	Beckman Coulter	Cat #A07789
D25 (anti-RSV F)	McLellan et al., 2013a	N/A
MPE8 (anti-RSV F)	Corti et al., 2013	N/A
Palivizumab (anti-RSV F)	Corti et al., 2013	N/A
AM14 (anti-RSV F)	Kwakkenbos et al., 2010	N/A

**Bacterial and Virus Strains**		

Human Respiratory Syncytial Virus with Green Fluorescent Protein. A2 strain	ViraTree	https://www.viratree.com/product/rsv-gfp1/

**Chemicals, Peptides, and Recombinant Proteins**		

DS-Cav1	McLellan et al., 2013a	N/A
RSV F (post fusion)	Corti et al., 2013	N/A
DS-Cav1–I53-50A	This paper	N/A
I53-50A	Bale et al., 2016	N/A
I53-50B.4PT1	Bale et al., 2016	N/A
Recombinant human interleukin-2	BD Biosciences	Cat #554603
D-desthiobiotin	Millipore Sigma	Cat #71610
Imidazole	Millipore Sigma	Cat #I5513
MEM, GlutaMAX Supplement	Thermo Fisher Scientific	Cat #41090028
Penicillin-Streptomycin	Thermo Fisher Scientific	Cat #10378016
Fetal Bovine Serum (FBS)	Thermo Fisher Scientific	Cat #10500064
Expi293 Expression Medium	Thermo Fisher Scientific	Cat #A1435101
Polyethylenimine (PEI)	Polysciences	Cat #24765

**Deposited Data**		

Single-particle cryoEM reconstruction of DS-Cav1–I53-50	This paper	EMDB: EMD-0350

**Experimental Models: Organisms/Strains**		

Mouse: BALB/c: (BALB/cOlaHsd)	Envigo	N/A

**Experimental Models: Cell Lines**		

Expi293F cells	ThermoFisher	Cat #A14527
HEK293T cells	ATCC	CRL-3216
HEp-2	ATCC	CCL-23
Mouse: primary T lymphocytes	This paper	N/A
Mouse: primary B lymphocytes	This paper	N/A

**Recombinant DNA**		

DS-Cav1	McLellan et al., 2013a	N/A
RSV F (post fusion)	Corti et al., 2013	N/A
pcDNA3.1 expression vector	Thermo Fisher Scientific	Cat #V79020

**Software and Algorithms**		

FlowJo v10	FlowJo, LLC	https://www.flowjo.com/solutions/flowjo
BD AttoVision	BD Biosciences	https://www.bdbiosciences.com/us/home
GraphPad Prism 7	GraphPad	https://www.graphpad.com/
Proteon Manager	Biorad	http://www.bio-rad.com/en-ch/product/proteon-manager-software?ID=49291a09-2c4c-4e56-a667-dba159e95684
Sic_axle	This paper	N/A

**Other**		

Strep-Tactin Superflow Agarose	Millipore Sigma	Cat #71592
HiTrap Protein G HP	GE Healthcare	Cat #17040501
Superose 6 3.2/300	GE Healthcare	Cat # 29036226
Zeba Spin Desalting Columns	Thermo Fisher Scientific	Cat #89882
Ni-NTA agarose	QIAGEN	Cat #30210
HiTrap MabSelect column	GE Healthcare	Cat #28408256
Superdex 200 16/600 gel filtration column	GE Healthcare	Cat #28989335
ProteOn GLC sensor chip	Biorad	Cat #1765011
Nunc-Immuno MicroWell 96 well solid plates	Millipore Sigma	Cat #M5785-1CS

### Contacts for Reagent and Resource Sharing

Further information and requests for reagents may be directed to the Lead Contact, Neil King (neilking@uw.edu).

### Experimental Model and Subject Details

#### Cell Lines

Expi293F cells, a female human embryonic kidney cell line adapted to grow in suspension, were obtained from Thermo Fisher Scientific. HEK293T cells were obtained from ATCC (CRL-3216). HEp-2 cells were also obtained from ATCC (CCL-23).

#### Mice

Female BALB/c mice 6–9 weeks old were obtained from Envigo (Italy). Animal procedures were performed in accordance with the guidelines of the Swiss Federal Veterinary Office and after obtaining ethical approval from the Ufficio Veterinario Cantonale, Bellinzona, Switzerland (approval number 332016).

#### Indian Rhesus Macaques

The study was approved by the Local Ethical Committee on Animal Experiments under the Swedish Board of Agriculture. The nine male Indian rhesus macaques were housed in the Astrid Fagraeus laboratory at Karolinska Institutet according to the guidelines of the Association for Assessment and Accreditation of Laboratory Animal Care, and all procedures were performed according to the provisions and general guidelines of the Swedish Animal Welfare Agency.

### Methods Details

#### Design of DS-Cav1–Bearing Nanoparticle Components

A custom docking protocol, sic_axle, was written in C++ to dock two proteins along a shared symmetry axis. The protocol was based on the two-component docking protocol originally described in (King et al., 2014). The protocol minimizes the distance between the two oligomeric proteins, and optionally the distance between user-specified termini (*d* in Figure 1A), while preventing clashes, defined as interatomic distances below a user-defined distance threshold. The degrees of freedom searched during docking are the displacement along the shared symmetry axis of one of the two proteins (*r*) and its rotation about the symmetry axis (ω). Crystal structures of DS-Cav1 (PDB: 4MMU) and DS-Cav1-foldon (PDB: 4MMV) (McLellan et al., 2013b) were docked against the computational design models of trimeric components from a set of designed self-assembling protein nanomaterials with exterior-facing termini. Docking results were inspected manually, and flexible genetic linkers were designed for promising antigen-nanoparticle component pairs. Images of design models were rendered using PyMOL 1.8.4.0 (Schrödinger, 2015).

#### Small-scale Expression and Screening

Genes encoding DS-Cav1 genetically fused to the N termini of nanoparticle components I3-01 (+/− foldon) and I53-50A (+ foldon) were synthesized and cloned by GenScript into pcDNA3.1 (I3-01) or pcDNA3+ (I53-50A) vectors. The remaining DS-Cav1 N-terminal fusions were cloned into pCMV (T33-15B ± foldon and T33-31A ± foldon; I32-28A 8GS or 12GS linker without foldon, I53-50A without foldon) using the KpnI and XhoI restriction sites. Amino acid sequences for all proteins used in this study are provided in Table S1. Plasmids were transformed into the NEB5α strain of *E. coli* (New England Biolabs) for subsequent DNA extraction from bacterial culture (NucleoBond® Xtra Midi kit) to obtain plasmid for transient transfection into Expi293F cells.

On day zero, Expi293F cells were seeded at a density 2.0 × 10^6^ cells/mL in Expi293 expression medium with 0.1 U/mL penicillin/streptomycin and incubated with 125 rpm oscillation at 37°C, 8% CO_2_, and 70% humidity. The following day, the culture density was adjusted to 2.5 × 10^6^ cells/mL, aliquoted into 12-well non-treated plates, and transfected using Life Technologies’ Expifectamine 293 Transfection kit in accordance with the manufacturer’s protocol. Enhancers were added on day 2, and cell suspensions were harvested on day 5 by centrifugation for 5 minutes at 4000 × g. The supernatants filtered through 0.45 μm PVDF filters.

For screening the clarified cell supernatants by ELISA, a standard curve was prepared by diluting trimeric DS-Cav1 to 160 μg/mL in Dulbecco’s phosphate buffered saline (dPBS) with 5% glycerol, making 4 × serial dilutions to a final concentration of 0.01 μg/mL and a total of 8 dilutions. All incubations were performed at room temperature with shaking for 1 h except where otherwise noted. 50 μL of clarified cell supernatants, standard curve dilutions, and I53-50A trimer at 10 μg/mL (negative control) were plated onto Pierce 96-well clear nickel-coated microplates (Thermo Fisher Scientific) and incubated. Plates were washed by submersion in wash buffer (25 mM Tris pH 8, 150 mM NaCl, 0.05% Tween 20) and forceful inversion six times. 200 μL of blocking buffer (wash buffer with 4% nonfat milk [Bio Rad]) was added to each well and incubated. The wash step was repeated. 0.2 μg/mL D25 primary antibody (see Expression and Purification of Monoclonal Antibodies) was added to each sample well and incubated. The wash step was repeated. 0.05 μg/mL of ab97160 horseradish peroxidase-conjugated anti-human secondary antibody (Abcam) was added to each well and incubated. The wash step was repeated. 150 μL of room temperature 1-Step ABTS Substrate Solution (Thermo Fisher Scientific) was added to each sample well. Plates were incubated for 15 minutes at room temperature with shaking until developed, the reaction was stopped with 100 μL of 1% SDS solution, and absorbance at 405 nm was measured in a SpectraMax M3 plate reader.

#### Protein Expression and Purification

The I53-50A trimer was expressed as described (Bale et al., 2016). Cells were lysed by sonication (2.5 minutes total sonicating time in 2 s pulses) in 50 mM Tris pH 8, 500 mM NaCl, 20 mM Imidazole, 0.75% 3-[(3-Cholamidopropyl)dimethylammonio]-1-propanesulfonate (CHAPS), 0.1 mg/mL lysozyme, 0.05 mg/mL DNase, 0.05 mg/mL RNase, 5 mM MgCl_2_ and 1 mM phenylmethylsulfonyl fluoride (PMSF). Lysate was clarified by centrifugation at 33,000 × g for 20 minutes. Lysate supernatants were applied to HisTrap HP or FF columns (GE Healthcare) for purification by immobilized metal affinity chromatography (IMAC) on an AKTA Pure FPLC system (GE Healthcare). Protein of interest was eluted over a linear gradient of 20 mM to 500 mM imidazole in a background of 50 mM Tris pH 8, 500 mM NaCl, and 0.75% CHAPS buffer after washing with ∼10 column volumes wash buffer (elution buffer with 20 mM imidazole). Peak fractions were pooled, concentrated in 10K MWCO centrifugal filters, sterile filtered (0.22 μm) and applied to a Superdex 200 Increase 10/300 GL SEC column (GE Healthcare) using 25 mM Tris pH 8, 500 mM NaCl, 0.75% CHAPS buffer, 1 mM DTT. I53-50A elutes at ∼15-17 mL. The I53-50B.4PT1 pentamer was expressed and purified as described (Bale et al., 2016).

DS-Cav1–I53-50A was produced by lentiviral transduction of HEK293F cells using the Daedalus system (Bandaranayake et al., 2011). Lentivirus was produced by transient transfection of HEK293T cells (ATCC) using linear 25 kDa polyethyleneimine (PEI; Polysciences). Briefly, 4 × 10^6^ cells were plated onto 10 cm tissue culture plates. After 24 h, 3 μg of psPAX2, 1.5 μg of pMD2G (Addgene plasmids #12260 and #12259, respectively), and 6 μg of lentiviral vector plasmid were mixed in 500 μL diluent (5 mM HEPES, 150 mM NaCl, pH 7.5) and 42 μL of PEI (1 mg/mL) and incubated for 15 minutes. The DNA/PEI complex was then added to the plate drop-wise. Lentivirus was harvested 48 h post-transfection and concentrated 100 × by centrifugation at 8000 × g for 18 h. Transduction of the target cell line was carried out in 125 mL shake flasks containing 10 × 10^6^ cells in 10 mL of growth media. 100 uL of 100 × lentivirus was added to the flask and the cells were incubated with 225 rpm oscillation at 37°C in 8% CO_2_ for 4–6 hours, after which 20 mL of growth media was added to the shake flask. Transduced cells were expanded every other day to a density of 1 × 10^6^ cells/mL until a final culture size of 4 L was reached. The media was harvested after 17 days of total incubation after measuring final cell concentration (∼5 × 10^6^ cells/mL) and viability (∼90% viable). Culture supernatant was harvested by low-speed centrifugation to remove cells from the supernatant. NaCl and NaN_3_ were added to final concentrations of 250 mM and 0.02%, respectively. The supernatant was loaded over one 5 mL HisTrap FF Crude column (GE Healthcare) at 5 mL/min by an AKTA Pure (GE Healthcare). The 5 mL HisTrap column was washed with 10 column volumes of wash buffer (2 × GIBCO 14200-075 PBS, 5 mM Imidazole, pH 7.5) followed by 6 column volumes of elution buffer (2 × GIBCO 14200-075 PBS, 150 mM Imidazole, pH 7.5). The nickel elution was applied to a HiLoad 16/600 Superdex 200 pg column (GE Healthcare) and run in dPBS (GIBCO 14190-144) with 5% glycerol (Thermo BP229-1) to further purify the target protein by SEC. The SEC-purified target protein was snap frozen in liquid nitrogen and stored at −80°C.

Postfusion RSV F and trimeric DS-Cav1 were produced as previously described (Corti et al., 2013, McLellan et al., 2013b). Expi293F cells were transfected with plasmids encoding DS-Cav1 or postfusion RSV F using polyethylenimine (PEI). Briefly, 1 μg plasmid/mL of cells (with cell density adjusted at 2.5 × 10^6^) and 10 μg PEI/mL of cells were diluted separately in OPTI-MEM (Thermo Fisher Scientific), mixed together, and incubated 15 minutes at room temperature (RT) before adding them to the cells. Transfected cells were cultured maintained for 7 days at 37°C with 8% CO_2_ and shaking at 135 rpm. Recombinant proteins were purified from cell supernatants by IMAC followed by affinity chromatography on Strep-Tactin superflow high capacity resin (IBA GmbH, Göttingen, Germany) using the C-terminal tandem Strep-tag. Proteins were eluted from the resin by competition with elution buffer (25 mM HEPES, pH 7.5, 150 mM NaCl) containing 5 mM desthiobiotin. The Strep-tag was cleaved by TEV protease (Thermo Fisher Scientific) treatment. Uncleaved protein and TEV protease were removed by negative IMAC chromatography. Finally, proteins were subjected to SEC on a Superdex 200 10/300 GL (GE Healthcare) equilibrated in PBS. The conformation of postfusion RSV F was confirmed by ELISA with palivizumab and D25.

#### Expression and Purification of Monoclonal Antibodies

Antibody (D25, MPE8 and AM14) heavy and light chains were ordered from GenScript and cloned into pcDNA3.1. Antibodies were expressed by transient co-transfection of both heavy and light chain plasmids in Expi293F cells using PEI (Polyscience). Cell supernatants were harvested after 7 days and passed over a HiTrap MabSelect column (GE Healthcare). Bound antibodies were washed with PBS and eluted with 100 mM glycine at pH 2.9 into 1/10th volume of 1 M Tris-HCl pH 8.0. Final purification of mAbs was performed by SEC on a Superdex 200 10/300 GL (GE Healthcare) using PBS as the mobile phase.

#### *In vitro* Assembly of DS-Cav1–I53-50 and I53-50

Concentrations of purified individual nanoparticle components were determined by measuring absorbance at 280 nm using a UV-Vis spectrophotometer (Agilent Cary 8454) and calculated extinction coefficients (Gasteiger et al., 2005). The following assembly steps were performed on ice: DS-Cav1–I53-50A trimer (in dPBS, 5% glycerol) and/or I53-50A trimer (in 25 mM Tris pH 8, 500 mM NaCl, 0.75% CHAPS) was added first to an eppendorf tube to a final concentration of 50 μM in the *in vitro* assembly reaction. Assembly buffer (25 mM Tris pH 8, 250 mM NaCl, 5% glycerol) was then added to a volume of 1 mL minus the total volumes of the components. Finally, I53-50B.4PT1 pentamer (in 25 mM Tris pH 8, 500 mM NaCl, 0.75% CHAPS) was added to the tube for a final concentration of 50 μM. In order to produce partial valency DS-Cav1–I53-50 nanoparticles (33% and 67% DS-Cav1–I53-50), DS-Cav1–I53-50A trimer was added to 16.7 μM (33% valency) or 33.3 μM (67% valency), and I53-50A trimer was added to 33.3 μM (33% valency) or 16.7 μM (67% valency). Assemblies were incubated at room temperature (I53-50 bare nanoparticle) or 4°C (DS-Cav1–I53-50 or partial valency nanoparticles) with gentle rocking for at least 1 hour before subsequent purification by SEC using a Superose 6 Increase 10/300 GL column. Assembled particles elute in the void volume of this column. Assembled nanoparticles were centrifuged for 10 minutes at 21000 × g and 4°C or sterile filtered (0.22 μm) immediately before column application.

#### Endotoxin Measurement and Removal

Endotoxin was removed from I53-50B.4PT1 during or after purification of the protein using a detergent wash during IMAC. The protein was immobilized on a 5 mL HisTrap HP column (GE Healthcare) equilibrated with buffer (25 mM Tris pH 8, 500 mM NaCl, 0.75% CHAPS) and the column was washed with ∼10 CV of the equilibration buffer. I53-50B.4PT1 was eluted with a linear gradient of 0 to 500 mM imidazole in equilibration buffer. Fractions containing I53-50B.4PT1 were concentrated in a 10 kDa molecular weight cutoff Vivaspin filter and dialyzed twice against equilibration buffer. Purified I53-50B.4PT1 pentamer was tested for endotoxin prior to assembly using a Charles River EndoSafe® PTS system, and measured concentrations were routinely below 100 EU/mL. Before formulation in SWE or AddaVax, protein preparations were again tested to be negative for endotoxin contamination by Chromogenic Limulus Amebocyte Lysate (LAL) assay (Lonza).

#### Analytical Size Exclusion Chromatography

Samples were sterile filtered (0.22 μm) or centrifuged for 10 minutes at 21000 × g and at 4°C immediately before column application. ∼3 mg of total protein was injected onto a Sephacryl S-500 HR 16/60 SEC column (GE Healthcare) on FPLC (AKTA Pure) using 25 mM Tris pH 8, 250 mM NaCl, 5% glycerol buffer as the mobile phase. I53-50 bare nanoparticle elutes ∼70-80 mL, DS-Cav1–I53-50 and partial valencies 60-70 mL, I53-50A trimeric component 80-90 mL, and I53-50B.4PT1 pentameric component 90-100 mL.

#### Dynamic Light Scattering

Triplicate measurements of 20 acquisitions each at 5 s per acquisition were taken on a DynaPro Nanostar instrument at 25°C in a 1 μL quartz cuvette (Wyatt Technology Corp.) and using auto-attenuation of the laser. Increased viscosity due to the inclusion of 5% glycerol in the DS-Cav1–I53-50 nanoparticles was accounted for in the software.

#### Negative Stain Electron Microscopy

For the negative stain EM appearing in Figures 1E, 3E, and 3F, I53-50 bare nanoparticle and DS-Cav1–I53-50 nanoparticles (including partial valency nanoparticles) were diluted to concentrations of 0.1 mg/mL and 0.05 mg/mL, respectively, in 25 mM Tris pH 8, 250 mM NaCl, 5% glycerol. We used carbon coated 300 mesh copper grids (Ted Pella), glow discharged immediately before use. 6 μL of sample was applied to the grid for 1 minute, then briefly dipped in a droplet of water before blotting away excess liquid with Whatman No. 1 filter paper. Grids were stained with 6 μL of 0.75% (w/v) uranyl formate stain, immediately blotting away excess, then stained again with another 6 μL for 30 s. Grids were imaged on a Morgagni transmission electron microscope with a Gatan camera. We used Gatan Digital Micrograph software to take images.

For the negative stain EM appearing in Figure S3, stock solution of DS-Cav1–I53-50 was diluted to an estimated concentration of 0.05 mg/mL in 25 mM Tris pH 8.0, 150 mM NaCl. We used carbon-coated Ted Pella G400 copper grids, glow discharged immediately before use. A volume of 3.5 μL of sample was deposited on the grid for 20-30 s before excess solution was blotted away using Whatman No. 1 filter paper. This was immediately followed by two rounds of staining using 3.5 μL of 2% (w/v) uranyl formate. Data were collected using an FEI Tecnai Spirit transmission electron microscope equipped with a Gatan US4000 CCD camera. Images were acquired with the Leginon software (Suloway et al., 2005) at a nominal magnification of 52,000 × at a defocus range comprised between −1 μm and −4 μm. CTF parameters were estimated using GCTF (Zhang, 2016). Particles were picked using DoG Picker (Voss et al., 2009). Particle images were extracted using a box size of 288 pixels binned by a factor of 2 to an effective pixel size of 4.14 Å and analyzed using RELION 2.1 (Kimanius et al., 2016). After 2 rounds of reference-free 2D classification, an initial model with icosahedral symmetry was generated from 2D class averages using the e2initialmodel.py function in EMAN2 (Tang et al., 2007). The 4,300 particles from the corresponding classes were used for 3D refinement of the initial model. The refined map was used as a reference for one additional round of 3D refinement to obtain the final map at an estimated resolution of 20 Å.

#### Cryo-electron Microscopy

Stock solution of DS-Cav1–I53-50 was applied directly on grids without further dilution. We used Protochips C-flat 1.2/1.3-4C-T carbon-coated copper grids, glow discharged immediately before use. A multiple blotting strategy was employed, as previously described (Snijder et al., 2017). After one round of sample application and blotting on the lab bench using Whatman No. 1 filter paper, a second volume of sample was applied to the grids, which were then mounted in an FEI Mark I Vitrobot for a final round of blotting and plunge-freezing in liquid ethane, using a 9 s blotting time with −3 mm offset at room temperature and 80%–90% relative humidity. Data was collected using the Leginon software (Suloway et al., 2005) on an FEI TF20 electron microscope, equipped with a Gatan K2 Summit direct electron detector. The dose rate was adjusted to 8 counts/pixel/s, and each movie was acquired in counting mode fractionated in 45 frames of 200 ms. 200 micrographs were collected in a single session with a defocus range comprised between −1.5 μm and −3.0 μm.

Movie frames were aligned with MotionCor2 (Zheng et al., 2017), with the use of dose weighting. CTF parameters were estimated from the aligned micrographs without applied dose weighting, using GCTF (Zhang, 2016). Particles were picked from aligned dose-weighted micrographs using DoG Picker (Voss et al., 2009). Particle images were extracted using a box size of 480 pixels binned by a factor of 2 to an effective pixel size of 2.5 Å and analyzed with RELION 2.0 (Kimanius et al., 2016). After 2 rounds of reference-free 2D classification, 1,600 particles were selected for 3D classification in 3 classes, starting with an initial model with icosahedral symmetry that was generated from 2D class averages using the e2initialmodel.py function in EMAN2 (Tang et al., 2007). One predominant class of 1,200 particles was selected to generate the final map, at 6.3 Å resolution, applying icosahedral symmetry, and using a solvent mask and the solvent_correct_fcs flag in RELION 2.0. A B-factor of −400 Å^2^ was applied to sharpen the map. Reported resolutions are based on the gold-standard FSC = 0.143 criterion (Rosenthal and Henderson, 2003) and Fourier shell correlation curves were corrected for the effects of soft masking by high-resolution noise substitution (Chen et al., 2013). ChimeraX was used for rendering (Goddard et al., 2018).

#### Surface Plasmon Resonance

For the retention of D25 binding after thermal stress, experiments were carried out at 20°C on a ProteON XPR-36 instrument (Bio-Rad Laboratories) in PBS (GIBCO, Thermo Fisher Scientific) and 0.05% Tween-20 (Sigma). D25 antibody was immobilized at 100 nM on a GLM sensor chip surface through amine coupling (EDC/NHS chemistry) and a blank surface with no antibody was created under identical coupling conditions for use as a reference. Analyte proteins (soluble DS-Cav1, soluble DS-Cav1–I53-50A, and DS-Cav1–I53-50 nanoparticle), heat stressed at various temperatures (20, 50, 70, and 80°C) for 1 h, were injected at a flow rate of 100 μL/minute at a concentration of 50 nM in the different sensor channels. Data were processed using Proteon Manager software and double-referenced by subtraction of the blank surface and buffer-only injection before local fitting of the data.

For the antigenic characterization by SPR, experiments were carried out at 20°C in PBS (GIBCO, Thermo Fisher Scientific) and 0.05% Tween-20 (Sigma). Monoclonal antibodies (mAbs) were immobilized at 100 nM on a GLM sensor chip surface through amine coupling (EDC/NHS chemistry) and a blank surface with no antibody was created under identical coupling conditions for use as a reference. Analyte proteins (soluble DS-Cav1 and DS-Cav1–I53-50 nanoparticles at various valencies) were injected at a flow rate of 100 μL/minute at several concentrations in the different sensor channels. Data were processed using Proteon Manager software and double-referenced by subtraction of the blank surface and buffer-only injection. *k*_*on*_, *k*_*off*_, and *K*_*D*_ were calculated by Langmuir fitting.

#### Bio-layer Interferometry

Binding of AM14 mAb to trimeric DS-Cav1 and DS-Cav1–I53-50 nanoparticles was analyzed using bio-layer interferometry with an Octet Red System (Pall FortéBio). Protein samples were diluted to 200 nM in kinetics buffer (HEPES-EP+ (FortéBio), with 0.05% nonfat milk). Buffer and sample were then applied to a black 96-well Greiner Bio-one microplate at 200 μL per well. Protein A biosensor tips (FortéBio) were first pre-wetted for 10 minutes in kinetics buffer, then the tips were dipped in mAb diluted to 10 μg/mL in kinetics buffer to load the biosensors, or buffer as a control. After 600 s, the tips were moved into buffer to reach baseline for another 120 s. The association step was performed by dipping the loaded tips into the protein samples for 500 s, then the dissociation was measured by dipping the tips back into fresh buffer for 1000 s. Plotted values are taken from 500 s into the dissociation step.

#### Guanidine Denaturation and Fluorescence

Trimeric DS-Cav1, DS-Cav1–I53-50A trimer, DS-Cav1–I53-50 nanoparticle, I53-50 bare nanoparticle, I53-50A trimer, or I53-50B pentamer was diluted to a final concentration of 2.5 μM in 25 mM Tris pH 8, 250 mM NaCl, 5% glycerol with [GdnHCl] (except I53-50B pentamer, which also included 0.75% CHAPS in the buffer) ranging from 0 M to 6.5 M, increasing in 0.25 M increments and prepared in triplicate. Samples were incubated for 16 hours at ambient temperature. A Peltier was used in the cell holder to maintain a temperature of 25°C throughout data collection. Using a Cary Eclipse Fluorescence Spectrophotometer and a 10 mm cell (Agilent Cuvette, part #6610021600), fluorescence spectra were collected, exciting at 290 nm and scanning from 310 nm to 510 nm at a rate of 60 nm/minute in 1 nm intervals with a bandpass of 1 nm.

#### Hydrogen/Deuterium-Exchange Mass Spectrometry

For each time point, 66 pmol of DS-Cav1 and DS-Cav1–I53-50 were incubated in deuterated buffer (85% D20, pH^∗^ 8.0) for 7, 60, 1,800, or 72,000 s at room temperature and subsequently mixed with an equal volume of ice-cold quench buffer (200 mM tris(2-chlorethyl) phosphate (TCEP), 0.2% formic acid) to a final pH of 2.5. Samples were immediately frozen in liquid nitrogen and stored at −80°C until analysis. Zero time point and fully deuterated samples were prepared as previously described (Verkerke et al., 2016). Online pepsin digestion was performed and analyzed by LC-MS-IMS utilizing a Waters Synapt G2-Si Q-TOF mass spectrometer as previously described (Verkerke et al., 2016). Deuterium uptake analysis was performed with HX-Express 3v14.2 (Guttman et al., 2013, Weis et al., 2006). The percent exchange was normalized to the zero time point and fully deuterated samples. Internal exchange standards (Pro-Pro-Pro-Ile [PPPI] and Pro-Pro-Pro-Phe [PPPF]) were included in each reaction to control for variations in ambient temperature during the labeling reactions.

#### Antigen Content Quantification

Before immunization of mice and NHPs, total protein concentration was measured using absorbance at 280 nm and calculated extinction coefficients as well as bicinchoninic acid (BCA) assay (Thermo Scientific) using bovine serum albumin as a standard for protein concentration determination.

#### Immunizations (Mice)

Female BALB/c mice 6–9 weeks old were obtained from Envigo (Italy). All proteins were formulated in PBS in a 1:1 ratio with AddaVax adjuvant (InvivoGen) according to the manufacturer’s instructions. For unadjuvanted immunizations, proteins were formulated in PBS and directly used for injection. Mice were immunized subcutaneously (s.c.) with a total protein dose corresponding to 5 μg of the DS-Cav1 antigen on day 0, 14, and 28. For the pre-immunization experiment presented in Figures 5D and 5E, the animals also received two injections of 5 μg unmodified I53-50 formulated in AddaVax on days −28 and −14. Mice were bled on day 24 and 38. Recovered sera were further used to measure binding and neutralizing titers.

#### Enzyme-Linked Immunosorbent Assay

Enzyme-linked immunosorbent assay (ELISA) was used to determine binding of sera and mAbs to the different proteins. Maxisorp (Nunc) ELISA plates were coated overnight at 4°C with 3 μg/mL of antigen. Plates were blocked with a 1% w/v solution of Bovine Serum Albumin (BSA; Sigma) in PBS for 1 hour at room temperature. Serial dilutions of mAbs or sera were added to the plates and, after washing, antibody binding was revealed using a goat anti-human IgG antibody coupled to alkaline phosphatase (Jackson Immunoresearch) for nonhuman primate sera and mAbs or with a goat anti-mouse IgG antibody coupled to alkaline phosphatase (Jackson Immunoresearch) for murine sera. Plates were then washed, substrate (p-NPP, Sigma) was added and absorbance was read at 405 nm.

#### Cells and Viruses

Expi293F cells were grown in Expi293 expression media, cultured at 37°C, 85% humidity, 8% CO_2,_ with shaking at 135 rpm. HEp-2 cells (ATCC® CCL-23), a female cell line derived from HeLa, were grown in Minimum Essential Medium (MEM) + GlutaMAX (Thermo Fisher Scientific) supplemented with 10% Fetal Bovine Serum (FBS) plus 100 IU/ml Penicillin/Streptomycin (Thermo Fisher Scientific) and cultured at 37°C, 5% CO_2_. HEp-2 cells were authenticated by analysis of Short Tandem Repeat (STR) loci at ATCC. Human respiratory syncytial virus with Green Fluorescent Protein, A2 strain, was obtained from ViraTree. All cell lines were confirmed to be free of Mycoplasma.

#### Virus Neutralization

Neutralization of RSV infection by mouse or NHP sera was measured using a micro-neutralization flow cytometry-based assay. Serial dilutions of sera were pre-incubated with RSV for 1 hour at 37°C and added to 10,000 HEp-2 cells/well in 96-well flat-bottom plates (MOI of 1). After 48 hours, cells were washed, detached and fixed with 2% formaldehyde. The percentage of GFP-positive cells was measured by high throughput FACS with an Intellicyt instrument coupled to an automated platform. The Tissue Culture Inhibiting Dilution neutralizing 50% of the infection (ID_50_) was calculated by nonlinear regression with Prism 7 (GraphPad Software).

#### Sera Immunodepletion

His-tagged depletion antigens (I53-50 or DS-Cav1) were immobilized on a HisTrap HP column (GE Healthcare) at 5 mg protein per mL resin using PBS as the mobile phase. Mice or NHP sera (100 μL diluted to 1 mL in PBS) were injected into the column and incubated for 30 minutes with immobilized antigen. Depleted sera were recovered by isocratic elution with PBS and further used for binding assays. Immobilized antibodies were recovered by acidic elution to ensure immunodepletion was successful.

#### Mice and Murine Lymphocyte Phenotyping

Mice were immunized subcutaneously (s.c.) with a total protein dose corresponding to 5 μg of DS-Cav1 antigen on day 0. All proteins were formulated in PBS in a 1:1 ratio with AddaVax adjuvant (InvivoGen). Six to seven mice per group were sacrificed at day 7 after priming, and draining lymph nodes were collected and converted to cell suspensions. The total number of cells in each suspension was counted by trypan blue exclusion with a hemocytometer. One-fifth of the cell suspension was used for FACS counting. The total number of Tfh and GC B cells (plotted in Figure 6) was calculated by multiplying the percentage of detected Tfh or GC B cells by the total number of cells in each suspension. The following antibodies were used to identify Tfh cells and GC B cells: CD4 (RM4-5), PD-1 (RMP1-30), B220 (RA3-6B2) (Thermo Fisher Scientific), CD3ε (17A2), CD45.1 (A20), ICOS (7E.17G9) (BioLegend), CXCR5 (2G8), CD19 (1D3), FAS (Jo2) (BD Biosciences), and Peanut Agglutinin (PNA) (Vector Laboratories). Dead cells were excluded from counting by staining with 7-aminoactinomycin D (7-AAD; BioLegend). Samples were acquired on a BD LSR Fortessa instrument and analyzed using the FlowJo software program (TreeStar).

#### Vaccine Formulation for NHP Immunizations

Squalene-in-water emulsion adjuvant (SWE) was prepared by the Vaccine Formulation Institute (VFI) as previously described (Ventura et al., 2013). 1 M Trizma hydrochloride, pH 8.0, 5 M sodium chloride, and glycerol were purchased from Sigma-Aldrich. Adjuvanted SWE-antigen formulations were prepared in PBS, without calcium or magnesium. TBS and glycerol were added to the formulation buffer used for DS-Cav1 soluble antigen formulation to mimic the composition of the buffer of DS-Cav1–I53-50 nanoparticles. Sodium chloride was added to all formulations to correct osmolarity. Average particle size (PS) was measured by DLS on a Zetasizer Nano ZS (Malvern), by backscattering at 173°. 10 μL of sample were diluted with 90 μL of citrate buffer. 70 μL were placed in a cuvette for PS measurement. Polydispersity index (PdI) was calculated based on PS measurements. Zeta potential was measured by electrophoretic light scattering on Zetasizer Nano ZS (Malvern, UK). 10 μL of sample were diluted with 990 μL of 1 mM NaCl in water. 1 mL was placed in a cuvette for zeta potential measurement. pH was measured by voltammetry on a pH meter (Seven Easy, Mettler Toledo) equipped with a microelectrode. The measurement was performed 3 times in a sample aliquot of 50 μL. Room temperature was regulated at 22°C. Squalene concentration was determined by reverse phase HPLC-UV at 208 nm. TEM analysis was performed on formulations containing DS-Cav1–I53-50 in SWE adjuvant after 2 weeks storage at 4°C. Formulations were either analyzed before or after centrifugation for 4 hours at 2100 × g to separate the aqueous phase from the oil phase. Samples (15 μL) were loaded on CANEMCO-MARIVAC carbon-coated 400 mesh copper grids treated by glow discharge. The grids were then placed on a drop of water, then on a drop of stain (2% uranyl acetate in water) for 30 s and finally they were let to dry on the bench at room temperature. The samples were imaged using a Tecnai 12 microscope and analyzed at 80 Kv.

#### NHP Immunizations and Sample Collection

Rhesus macaques were divided into two groups, age and weight matched, receiving either trimeric DS-Cav1 (n = 4) or DS-Cav1–I53-50 (n = 5) formulated in a squalene-based oil-in-water emulsion adjuvant (SWE; Vaccine Formulation Institute). A dose of 50 μg of DS-Cav1 antigen (96 μg total protein mass for DS-Cav1–I53-50) was administered intramuscularly in both groups at weeks 0 and 4. The animals were lightly sedated with ketamine at 10–15 mg/kg given intramuscularly (Ketaminol 100 mg/mL, Intervet, Sweden) during the immunizations and blood draws.

### Quantification and Statistical Analysis

No statistical methods were used to predetermine sample size. For mice samples analysis, no blinding of the experimenter was done. For NHP sera analysis, experimenters were blinded to the group appartenance of the animals analyzed. Statistical parameters including the exact value of n, the definition of center, dispersion, and precision measures (geometric mean ± SEM) and statistical significance are reported in the Figures and Figure Legends. Data were judged to be statistically significant when p < 0.05. In Figures, asterisks denote statistical significance as calculated using the two-tailed non-parametric Mann-Whitney U test for two groups’ comparison or one-Way ANOVA with multiple comparison corrected by Tukey’s test when three or more groups were compared. Analyses were performed in GraphPad PRISM 7.

### Data and Software Availability

The EMDB Accession Number for the single-particle cryoEM reconstruction of the DS-Cav1–I53-50 nanoparticle by cryo-electron microscopy is EMDB: EMD-0350. All software used in this study is listed in the Key Resources Table. A static executable of the sic_axle program used to dock DS-Cav1 to trimeric nanoparticle components is available upon request (neilking@uw.edu).

## Supplemental Information

Table S1. Amino Acid Sequences of Proteins Used in This Work, Related to Figures 1–7

Table S2. K_D_ and k_on_ Values Calculated from the SPR Data Presented in Figure S2C, Related to Figure 1
