# Supplementary material for: Induction of Potent Neutralizing Antibody Responses by a Designed Protein Nanoparticle Vaccine for Respiratory Syncytial Virus
Source: Cell. 2019 Mar 7;176(6):1420–1431.e17. doi: 10.1016/j.cell.2019.01.046 (PMC6424820; doi:10.1016/j.cell.2019.01.046)
Supplement: Table S1. Amino Acid Sequences of Proteins Used in This Work, Related to Figures 1–7 [file mmc1.pdf]

Cell, Volume *177*

Supplemental Information

Induction of Potent Neutralizing Antibody Responses by a Designed Protein Nanoparticle Vaccine for Respiratory Syncytial Virus

Jessica Marcandalli, Brooke Fiala, Sebastian Ols, Michela Perotti, Willem de van der Schueren, Joost Snijder, Edgar Hodge, Mark Benhaim, Rashmi Ravichandran, Lauren Carter, Will Sheffler, Livia Brunner, Maria Lawrenz, Patrice Dubois, Antonio Lanzavecchia, Federica Sallusto, Kelly K. Lee, David Veesler, Colin E. Correnti, Lance J. Stewart, David Baker, Karin Loré, Laurent Perez, and Neil P. King

Table S1. Amino acid sequences of proteins used in this work, related to Figures 1-7

>DS-Cav1 +foldon
MELLILKANAITTILTAVTFCFASGQNITEEFYQSTCSAVSKGYLSALRTGWYTSVITIELSNIKENKCNGTDAKVKLIKQELD
KYKNAVTELQLLMQSTPATNNRARRELPRFMNYTLNNAKKTNVTLSKKRKRRFLGFLLGVGSAIASGVAVCKVLHLEGE
VNKIKSALLSTNKAVVSLSNGVSVLTFKVLDLKNYIDKQLLPILNKQSCSISNIETVIEFQQKNNRLLEITREFSVNAGVTTPV
STYMLTNSELLSLINDMPITNDQKKLMSNNVQIVRQQSYSIMCIIKEEVLAYVVQLPLYGVIDTPCWKLHTSPLCTTNTKE
GSNICLTRTDRGWYCDNAGSVSFFPQAETCKVQSNRVFCDTMNSLTLPSEVNLCNVDIFNPKYDCKIMTSKTDVSSSVI
TSLGAIVSCYGKTKCTASNKNRGIIKTFSNGCDYVSNKGVDTVSVGNTLYYVNKQEGKSLYVKGEPIINFYDPLVFPSDEF
DASISQVNEKINQSLAFIRKSDELLSAIGGYIPEAPRDGQAYVRKDGEWVLLSTFLENLYFQSSAWSHPQFEKGGGSGGG
SGGSAWSHPQFEKGSGSGSGLNDIFEAQKIEWHEGSGSGSHHHHHHHH

>Postfusion RSV F -foldon
MELLILKANAITTILTAVTFCFASGQNITEEFYQSTCSAVSKGYLSALRTGWYTSVITIELSNIKKNKCNGTDAKIKLIKQELD
KYKNAVTELQLLMQSTPATNNRARRELPRFMNYTLNNAKKTNVTLSKKRKRRFLGFLLGVGSAIASGVAVSKVLHLEGE
VNKIKSALLSTNKAVVSLSNGVSVLTSKVLDLKNYIDKQLLPIVNKQSCSISNIETVIEFQQKNNRLLEITREFSVNAGVTTP
VSTYMLTNSELLSLINDMPITNDQKKLMSNNVQIVRQQSYSIMSIIKEEVLAYVVQLPLYGVIDTPCWKLHTSPLCTTNTK
EGSNICLTRTDRGWYCDNAGSVSFFPQAETCKVQSNRVFCDTMNSLTLPSEVNLCNVDIFNPKYDCKIMTSKTDVSSSV
ITSLGAIVSCYGKTKCTASNKNRGIIKTFSNGCDYVSNKGVDTVSVGNTLYYVNKQEGKSLYVKGEPIINFYDPLVFPSDEF
DASISQVNEKINQSLAFIRKSDELLHNVNAVENLYFQSSAWSHPQFEKGGGSGGGSGGSAWSHPQFEKGSGSGSGLN
DIFEAQKIEWHEGSGSGSHHHHHHHH

>DS-Cav1–I53-50A +foldon (“DS-Cav1–I53-50A”)
MELLILKANAITTILTAVTFCFASGQNITEEFYQSTCSAVSKGYLSALRTGWYTSVITIELSNIKENKCNGTDAKVKLIKQELD
KYKNAVTELQLLMQSTPATNNRARRELPRFMNYTLNNAKKTNVTLSKKRKRRFLGFLLGVGSAIASGVAVCKVLHLEGE
VNKIKSALLSTNKAVVSLSNGVSVLTFKVLDLKNYIDKQLLPILNKQSCSISNIETVIEFQQKNNRLLEITREFSVNAGVTTPV
STYMLTNSELLSLINDMPITNDQKKLMSNNVQIVRQQSYSIMCIIKEEVLAYVVQLPLYGVIDTPCWKLHTSPLCTTNTKE
GSNICLTRTDRGWYCDNAGSVSFFPQAETCKVQSNRVFCDTMNSLTLPSEVNLCNVDIFNPKYDCKIMTSKTDVSSSVI
TSLGAIVSCYGKTKCTASNKNRGIIKTFSNGCDYVSNKGVDTVSVGNTLYYVNKQEGKSLYVKGEPIINFYDPLVFPSDEF
DASISQVNEKINQSLAFIRGYIPEAPRDGQAYVRKDGEWVLLSTFLGSGSHHHHHHHHGGSGGSGSEKAAKAEEAARK
MEELFKKHKIVAVLRANSVEEAIEKAVAVFAGGVHLIEITFTVPDADTVIKALSVLKEKGAIIGAGTVTSVEQCRKAVESGA
EFIVSPHLDEEISQFCKEKGVFYMPGVMTPTELVKAMKLGHTILKLFPGEVVGPQFVKAMKGPFPNVKFVPTGGVNLD
NVCEWFKAGVLAVGVGSALVKGTPDEVREKAKAFVEKIRGCTE

>I53-50A
MKMEELFKKHKIVAVLRANSVEEAIEKAVAVFAGGVHLIEITFTVPDADTVIKALSVLKEKGAIIGAGTVTSVEQCRKAVE
SGAEFIVSPHLDEEISQFCKEKGVFYMPGVMTPTELVKAMKLGHTILKLFPGEVVGPQFVKAMKGPFPNVKFVPTGGV
NLDNVCEWFKAGVLAVGVGSALVKGTPDEVREKAKAFVEKIRGCTELEHHHHHH

>I53-50B.4PT1
MNQHSHKDHETVRIAVVRARWHAEIVDACVSAFEAAMRDIGGDRFAVDVFDVPGAYEIPLHARTLAETGRYGAVLGT
AFVVNGGIYRHEFVASAVINGMMNVQLNTGVPVLSAVLTPHNYDKSKAHTLLFLALFAVKGMEAARACVEILAAREKI
AAGSLEHHHHHH

>2obx-wt
MNQHSHKDYETVRIAVVRARWHADIVDQCVSAFEAEMADIGGDRFAVDVFDVPGAYEIPLHARTLAETGRYGAVLGT
AFVVNGGIYRHEFVASAVIDGMMNVQLSTGVPVLSAVLTPHNYHDSAEHHRFFFEHFTVKGKEAARACVEILAAREKIA
AGSLEHHHHHH

>DS-Cav1–I53-50A -foldon
MELLILKANAITTILTAVTFCFASGQNITEEFYQSTCSAVSKGYLSALRTGWYTSVITIELSNIKENKCNGTDAKVKLIKQELD
KYKNAVTELQLLMQSTPATNNRARRELPRFMNYTLNNAKKTNVTLSKKRKRRFLGFLLGVGSAIASGVAVCKVLHLEGE
VNKIKSALLSTNKAVVSLSNGVSVLTFKVLDLKNYIDKQLLPILNKQSCSISNIETVIEFQQKNNRLLEITREFSVNAGVTTPV
STYMLTNSELLSLINDMPITNDQKKLMSNNVQIVRQQSYSIMCIIKEEVLAYVVQLPLYGVIDTPCWKLHTSPLCTTNTKE
GSNICLTRTDRGWYCDNAGSVSFFPQAETCKVQSNRVFCDTMNSLTLPSEVNLCNVDIFNPKYDCKIMTSKTDVSSSVI
TSLGAIVSCYGKTKCTASNKNRGIIKTFSNGCDYVSNKGVDTVSVGNTLYYVNKQEGKSLYVKGEPIINFYDPLVFPSDEF
DASISQVNEKINQSLAFIRGGSGGSGSEKAAKAEEAARKMEELFKKHKIVAVLRANSVEEAIEKAVAVFAGGVHLIEITFT
VPDADTVIKALSVLKEKGAIIGAGTVTSVEQCRKAVESGAEFIVSPHLDEEISQFCKEKGVFYMPGVMTPTELVKAMKLG
HTILKLFPGEVVGPQFVKAMKGPFPNVKFVPTGGVNLDNVCEWFKAGVLAVGVGSALVKGTPDEVREKAKAFVEKIRG
CTEGSHHHHHH

>DS-Cav1–I3-01 +foldon
MELLILKANAITTILTAVTFCFASGQNITEEFYQSTCSAVSKGYLSALRTGWYTSVITIELSNIKENKCNGTDAKVKLIKQELD
KYKNAVTELQLLMQSTPATNNRARRELPRFMNYTLNNAKKTNVTLSKKRKRRFLGFLLGVGSAIASGVAVCKVLHLEGE
VNKIKSALLSTNKAVVSLSNGVSVLTFKVLDLKNYIDKQLLPILNKQSCSISNIETVIEFQQKNNRLLEITREFSVNAGVTTPV
STYMLTNSELLSLINDMPITNDQKKLMSNNVQIVRQQSYSIMCIIKEEVLAYVVQLPLYGVIDTPCWKLHTSPLCTTNTKE
GSNICLTRTDRGWYCDNAGSVSFFPQAETCKVQSNRVFCDTMNSLTLPSEVNLCNVDIFNPKYDCKIMTSKTDVSSSVI
TSLGAIVSCYGKTKCTASNKNRGIIKTFSNGCDYVSNKGVDTVSVGNTLYYVNKQEGKSLYVKGEPIINFYDPLVFPSDEF
DASISQVNEKINQSLAFIRGYIPEAPRDGQAYVRKDGEWVLLSTFLGGSGGSGSEKAAKAEEAARKMEELFKKHKIVAVL
RANSVEEAKKKALAVFLGGVHLIEITFTVPDADTVIKELSFLKEMGAIIGAGTVTSVEQCRKAVESGAEFIVSPHLDEEISQF
CKEKGVFYMPGVMTPTELVKAMKLGHTILKLFPGEVVGPQFVKAMKGPFPNVKFVPTGGVNLDNVCEWFKAGVLAV
GVGSALVKGTPVEVAEKAKAFVEKIRGCTEGSENLYFQGSGHHHHHHGSGSGWSHPQFEKGSGSWSHPQFEKSR

>DS-Cav1–I3-01 -foldon
MELLILKANAITTILTAVTFCFASGQNITEEFYQSTCSAVSKGYLSALRTGWYTSVITIELSNIKENKCNGTDAKVKLIKQELD
KYKNAVTELQLLMQSTPATNNRARRELPRFMNYTLNNAKKTNVTLSKKRKRRFLGFLLGVGSAIASGVAVCKVLHLEGE
VNKIKSALLSTNKAVVSLSNGVSVLTFKVLDLKNYIDKQLLPILNKQSCSISNIETVIEFQQKNNRLLEITREFSVNAGVTTPV
STYMLTNSELLSLINDMPITNDQKKLMSNNVQIVRQQSYSIMCIIKEEVLAYVVQLPLYGVIDTPCWKLHTSPLCTTNTKE
GSNICLTRTDRGWYCDNAGSVSFFPQAETCKVQSNRVFCDTMNSLTLPSEVNLCNVDIFNPKYDCKIMTSKTDVSSSVI
TSLGAIVSCYGKTKCTASNKNRGIIKTFSNGCDYVSNKGVDTVSVGNTLYYVNKQEGKSLYVKGEPIINFYDPLVFPSDEF
DASISQVNEKINQSLAFIRGGSGGSGSEKAAKAEEAARKMEELFKKHKIVAVLRANSVEEAKKKALAVFLGGVHLIEITFT
VPDADTVIKELSFLKEMGAIIGAGTVTSVEQCRKAVESGAEFIVSPHLDEEISQFCKEKGVFYMPGVMTPTELVKAMKLG
HTILKLFPGEVVGPQFVKAMKGPFPNVKFVPTGGVNLDNVCEWFKAGVLAVGVGSALVKGTPVEVAEKAKAFVEKIRG
CTEGSENLYFQGSGHHHHHHGSGSGWSHPQFEKGSGSWSHPQFEKSR

>DS-Cav1–T33-15B +foldon
MELLILKANAITTILTAVTFCFASGQNITEEFYQSTCSAVSKGYLSALRTGWYTSVITIELSNIKENKCNGTDAKVKLIKQELD
KYKNAVTELQLLMQSTPATNNRARRELPRFMNYTLNNAKKTNVTLSKKRKRRFLGFLLGVGSAIASGVAVCKVLHLEGE

VNKIKSALLSTNKAVVSLSNGVSVLTFKVLDLKNYIDKQLLPILNKQSCSISNIETVIEFQQKNNRLLEITREFSVNAGVTTPV
STYMLTNSELLSLINDMPITNDQKKLMSNNVQIVRQQSYSIMCIIKEEVLAYVVQLPLYGVIDTPCWKLHTSPLCTTNTKE
GSNICLTRTDRGWYCDNAGSVSFFPQAETCKVQSNRVFCDTMNSLTLPSEVNLCNVDIFNPKYDCKIMTSKTDVSSSVI
TSLGAIVSCYGKTKCTASNKNRGIIKTFSNGCDYVSNKGVDTVSVGNTLYYVNKQEGKSLYVKGEPIINFYDPLVFPSDEF
DASISQVNEKINQSLAFIRKSDELLGYIPEAPRDGQAYVRKDGEWVLLSTFLGGSMVRGIRGAITVNSDTPTSIIIATILLLE
KMLEANGIQSYEELAAVIFTVTEDLTSAFPAEAARQIGMHRVPLLSAREVPVPGSLPRVIRVLALWNTDTPQDRVRHVYL
SEAVRLRPDLESAQGSHHHHHH

>DS-Cav1–T33-15B -foldon
MELLILKANAITTILTAVTFCFASGQNITEEFYQSTCSAVSKGYLSALRTGWYTSVITIELSNIKENKCNGTDAKVKLIKQELD
KYKNAVTELQLLMQSTPATNNRARRELPRFMNYTLNNAKKTNVTLSKKRKRRFLGFLLGVGSAIASGVAVCKVLHLEGE
VNKIKSALLSTNKAVVSLSNGVSVLTFKVLDLKNYIDKQLLPILNKQSCSISNIETVIEFQQKNNRLLEITREFSVNAGVTTPV
STYMLTNSELLSLINDMPITNDQKKLMSNNVQIVRQQSYSIMCIIKEEVLAYVVQLPLYGVIDTPCWKLHTSPLCTTNTKE
GSNICLTRTDRGWYCDNAGSVSFFPQAETCKVQSNRVFCDTMNSLTLPSEVNLCNVDIFNPKYDCKIMTSKTDVSSSVI
TSLGAIVSCYGKTKCTASNKNRGIIKTFSNGCDYVSNKGVDTVSVGNTLYYVNKQEGKSLYVKGEPIINFYDPLVFPSDEF
DASISQVNEKINQSLAFIRKSDELLGGSMVRGIRGAITVNSDTPTSIIIATILLLEKMLEANGIQSYEELAAVIFTVTEDLTSAF
PAEAARQIGMHRVPLLSAREVPVPGSLPRVIRVLALWNTDTPQDRVRHVYLSEAVRLRPDLESAQGSHHHHHH

>DS-Cav1–T33-31A +foldon
MELLILKANAITTILTAVTFCFASGQNITEEFYQSTCSAVSKGYLSALRTGWYTSVITIELSNIKENKCNGTDAKVKLIKQELD
KYKNAVTELQLLMQSTPATNNRARRELPRFMNYTLNNAKKTNVTLSKKRKRRFLGFLLGVGSAIASGVAVCKVLHLEGE
VNKIKSALLSTNKAVVSLSNGVSVLTFKVLDLKNYIDKQLLPILNKQSCSISNIETVIEFQQKNNRLLEITREFSVNAGVTTPV
STYMLTNSELLSLINDMPITNDQKKLMSNNVQIVRQQSYSIMCIIKEEVLAYVVQLPLYGVIDTPCWKLHTSPLCTTNTKE
GSNICLTRTDRGWYCDNAGSVSFFPQAETCKVQSNRVFCDTMNSLTLPSEVNLCNVDIFNPKYDCKIMTSKTDVSSSVI
TSLGAIVSCYGKTKCTASNKNRGIIKTFSNGCDYVSNKGVDTVSVGNTLYYVNKQEGKSLYVKGEPIINFYDPLVFPSDEF
DASISQVNEKINQSLAFIRGYIPEAPRDGQAYVRKDGEWVLLSTFLGSGSMEEVVLITVPSALVAVKIAHALVEERLAACV
NIVPGLTSIYREEGSVVSDHELLLLVKTTTDAFPKLKERVKELHPYEVPEIVALPIAEGNREYLDWLRENTENLYFQGQKLIS
EEDLHHHHHH

>DS-Cav1–T33-31A -foldon
MELLILKANAITTILTAVTFCFASGQNITEEFYQSTCSAVSKGYLSALRTGWYTSVITIELSNIKENKCNGTDAKVKLIKQELD
KYKNAVTELQLLMQSTPATNNRARRELPRFMNYTLNNAKKTNVTLSKKRKRRFLGFLLGVGSAIASGVAVCKVLHLEGE
VNKIKSALLSTNKAVVSLSNGVSVLTFKVLDLKNYIDKQLLPILNKQSCSISNIETVIEFQQKNNRLLEITREFSVNAGVTTPV
STYMLTNSELLSLINDMPITNDQKKLMSNNVQIVRQQSYSIMCIIKEEVLAYVVQLPLYGVIDTPCWKLHTSPLCTTNTKE
GSNICLTRTDRGWYCDNAGSVSFFPQAETCKVQSNRVFCDTMNSLTLPSEVNLCNVDIFNPKYDCKIMTSKTDVSSSVI
TSLGAIVSCYGKTKCTASNKNRGIIKTFSNGCDYVSNKGVDTVSVGNTLYYVNKQEGKSLYVKGEPIINFYDPLVFPSDEF
DASISQVNEKINQSLAFIRGSGSMEEVVLITVPSALVAVKIAHALVEERLAACVNIVPGLTSIYREEGSVVSDHELLLLVKTT
TDAFPKLKERVKELHPYEVPEIVALPIAEGNREYLDWLRENTENLYFQGQKLISEEDLHHHHHH

>DS-Cav1–8GS–I32-28A
MELLILKANAITTILTAVTFCFASGQNITEEFYQSTCSAVSKGYLSALRTGWYTSVITIELSNIKENKCNGTDAKVKLIKQELD
KYKNAVTELQLLMQSTPATNNRARRELPRFMNYTLNNAKKTNVTLSKKRKRRFLGFLLGVGSAIASGVAVCKVLHLEGE
VNKIKSALLSTNKAVVSLSNGVSVLTFKVLDLKNYIDKQLLPILNKQSCSISNIETVIEFQQKNNRLLEITREFSVNAGVTTPV
STYMLTNSELLSLINDMPITNDQKKLMSNNVQIVRQQSYSIMCIIKEEVLAYVVQLPLYGVIDTPCWKLHTSPLCTTNTKE

GSNICLTRTDRGWYCDNAGSVSFFPQAETCKVQSNRVFCDTMNSLTLPSEVNLCNVDIFNPKYDCKIMTSKTDVSSSVI
TSLGAIVSCYGKTKCTASNKNRGIIKTFSNGCDYVSNKGVDTVSVGNTLYYVNKQEGKSLYVKGEPIINFYDPLVFPSDEF
DASISQVNEKINQSLAFIRKSDELLGGSGGSGSGDDARIAAIGDVDELNSQIGVLLAEPLPDDVRAALSAIQHDLFDLGGE
LCIPGHAAITEDHLLRLALWLVHYNGQLPPLEEFILPGGARGAALAHVCRTVCRRAERSIKALGASEPLNIAPAAYVNLLS
DLLFVLARVLNRAAGGADVLWDRTRAHGSHHHHHH

>DS-Cav1–12GS–I32-28A
MELLILKANAITTILTAVTFCFASGQNITEEFYQSTCSAVSKGYLSALRTGWYTSVITIELSNIKENKCNGTDAKVKLIKQELD
KYKNAVTELQLLMQSTPATNNRARRELPRFMNYTLNNAKKTNVTLSKKRKRRFLGFLLGVGSAIASGVAVCKVLHLEGE
VNKIKSALLSTNKAVVSLSNGVSVLTFKVLDLKNYIDKQLLPILNKQSCSISNIETVIEFQQKNNRLLEITREFSVNAGVTTPV
STYMLTNSELLSLINDMPITNDQKKLMSNNVQIVRQQSYSIMCIIKEEVLAYVVQLPLYGVIDTPCWKLHTSPLCTTNTKE
GSNICLTRTDRGWYCDNAGSVSFFPQAETCKVQSNRVFCDTMNSLTLPSEVNLCNVDIFNPKYDCKIMTSKTDVSSSVI
TSLGAIVSCYGKTKCTASNKNRGIIKTFSNGCDYVSNKGVDTVSVGNTLYYVNKQEGKSLYVKGEPIINFYDPLVFPSDEF
DASISQVNEKINQSLAFIRKSDELLGGSGGSGSGSGSDDARIAAIGDVDELNSQIGVLLAEPLPDDVRAALSAIQHDLFDL
GGELCIPGHAAITEDHLLRLALWLVHYNGQLPPLEEFILPGGARGAALAHVCRTVCRRAERSIKALGASEPLNIAPAAYVN
LLSDLLFVLARVLNRAAGGADVLWDRTRAHGSHHHHHH
