# Supplementary material for: Induction of Potent Neutralizing Antibody Responses by a Designed Protein Nanoparticle Vaccine for Respiratory Syncytial Virus
Source: Cell. 2019 Mar 7;176(6):1420–1431.e17. doi: 10.1016/j.cell.2019.01.046 (PMC6424820; doi:10.1016/j.cell.2019.01.046)
Supplement: Table S2. KD and kon Values Calculated from the SPR Data Presented in Figure S2C, Related to Figure 1 [file mmc2.pdf]

Supplementary Table 2. ***K_D* and *k_{on}* values calculated from the SPR data presented in Figure S2C, Related to Figure 1**

	K_D (M)		k_{on} (M^{-1} s^{-1})				
mAb	DS-Cav1	DS-Cav1–I53-50A	DS-Cav1	DS-Cav1–I53-50A	33% DS-Cav1–I53-50	67% DS-Cav1–I53-50	100% DS-Cav1–I53-50
D25	3.1×10^{-10}	4.7×10^{-10}	4.5×10^{5}	3.7×10^{5}	1.4×10^{5}	2.0×10^{5}	1.7×10^{5}
MPE8	1.1×10^{-10}	2.0×10^{-10}	4.2×10^{5}	4.4×10^{5}	2.0×10^{5}	3.9×10^{5}	3.9×10^{5}
AM14	7.8×10^{-11}	1.8×10^{-10}	5.5×10^{5}	3.6×10^{5}	4.0×10^{5}	4.1×10^{5}	5.9×10^{5}
